# A mixed-methods investigation for effects of built environments on older people’s social interaction in care homes

**DOI:** 10.3389/fpubh.2025.1693935

**Published:** 2025-10-29

**Authors:** Chendi Wang, Yujian Pan, Xingjian Li, Shaohua Qiang

**Affiliations:** Department of Construction Management and Real Estate, School of Economics and Management, Nanjing Tech University, Nanjing, China

**Keywords:** built environment, older people, social interaction, care homes, mixed-methods investigation

## Abstract

This study examines relationships between the built environment (BE) and social interaction (SI) among older adults in care homes. A mixed-methods design combined questionnaires (*n =* 119), environmental measurements, and behavioral observations across three care homes. An integrated BE-SI model, developed using multivariate statistical analyses, identified key environmental determinants of SI. Results indicate that recreational spaces, lighting, functional facilities, and accessibility significantly influenced interpersonal interactions, activity engagement, resident-caregiver relations, and conflict. Objective environmental measures corroborated survey and observational findings: care homes with larger recreational areas and improved functional accessibility showed higher frequencies of resident social engagement. These findings highlight the critical role of environmental design in promoting social participation among older residents. The study offers evidence-based recommendations for designers, facility managers, and policymakers to create age-friendly care-home environments that foster social interaction and enhance residents’ wellbeing. The BE-SI model provides a practical framework for future research, facility evaluation, and policy implementation.

## 1 Introduction

The global aging population demands urgent enhancement of institutional care environments. By 2050, one in six people worldwide will be over 65 (1), with China projected to have 400 million older adults by 2040 (2). Care homes, serving older adults with physical or cognitive impairments (3), face critical challenges. Despite ensuring safety and professional care, they often disrupt residents’ social networks. Residents experience a 40–60% reduction in meaningful social interactions (SI) compared to peers aging in place (4), leading to “relocation shock” marked by broken community ties and limited mobility (5, 6). Unlike community settings where SI arise naturally, care homes impose constraints through physical limitations and institutional routines, resulting in interactions dominated by structured activities and caregiver mediation (7, 8). These occur within built environments (BE) prioritizing operational efficiency over social facilitation (9). This study explores residents’ subjective experiences of environmental factors affecting SI. By emphasizing lived perceptions over architectural metrics (10–12), it offers design recommendations to balance functional and social needs, guiding care homes toward social sustainability.

## 2 Literature review

The relationship between older adults’ social lives and their physical surroundings is powerfully explained by the Person-Environment (P-E) fit theory. This foundational theory posits that wellbeing and adaptive behavior depend on the congruence between an individual’s personal characteristics (P)—such as their needs, abilities, and preferences—and their external environment (E), which includes its demands and resources (13–15). A strong P-E fit in care homes is thus essential for fostering positive social outcomes and mitigating the challenges of aging.

### 2.1 Social interaction of older people

As people age, their social interaction abilities diminish due to cognitive decline, physical limitations, and sensory impairments (16, 17). This decline is more pronounced in care homes, leading to increased social isolation (5, 18). *Interpersonal interactions*, including group conversations, casual dialogues, and resident visits, are vital for maintaining social bonds (19–21), enhancing emotional wellbeing, and reducing loneliness (11, 12, 22). However, the frequency and quality of these interactions may be significantly influenced by the environment (23). *Activity engagement* in care homes presents unique characteristics shaped by aging-related limitations, institutional environments, and social dynamics. Most residents require mobility aids for participation and demonstrate higher dependence on structured group activities compared to community-dwelling peers (24). Centralized activity hubs and circular seating arrangements increase spontaneous communication (25).

*Caregiver relationships* are crucial for social interactions and wellbeing in care facilities (26, 27). These relationships provide social support and reduce loneliness (28, 29). Well-designed layouts that minimize physical barriers improve caregiver-resident interactions (30). Positive relationships provide emotional support, enhancing self-confidence and encouraging interaction initiation (31). *Conflict* in care homes is intertwined with built environment design. Environmental conditions shape conflict frequency, intensity, and resolution (32). Poorly designed recreational areas may heighten interpersonal friction by limiting residents’ autonomy (33). Conversely, intentional design with well-zoned spaces for quiet relaxation versus group activities reduces sensory overload (34), while functional layouts separating high-traffic zones from private retreats help manage group dynamics (35).

### 2.2 Built environment in care homes

In care homes, the built environment (BE), encompassing distinct spaces, buildings, and surroundings, serves as the primary living area for older adults (36, 37). *Space* design fundamentally influences residents’ mobility and social interaction (SI) (26, 38). While narrow spaces may restrict movement and heighten territorial disputes (34). *Recreational areas* designed with accessibility act as hubs for physical and social participation (39, 78). Spacious entrances and wide passageways encourage involvement in cultural activities (40), while well-planned zones mitigate overcrowding. Furniture and equipment arrangement directly impacts SI. Circular seating *layouts* foster face-to-face interactions and group cohesion (41), whereas haphazard placements fragment social groups or hinder supervision (42). *Daylight*-filled areas enhance visual clarity and uplift moods, while poorly lit environments may induce anxiety, particularly for those with cognitive impairments (43, 44). *Distance* between residential units and recreational zones shapes participation willingness, with shorter pathways encouraging frequent use of communal spaces (45).

Diverse *facilities* support varied activity engagements, with well-equipped rooms enabling group exercises and fostering collaboration. Inadequate facilities may limit options, leading to boredom (46). This is a classic example of poor P-E fit, where the environment fails to provide the resources needed to meet residents’ social and recreational needs. *Barrier-free* design principles ensure equitable access to SI, empowering residents’ autonomy and reducing dependency-related tensions (32). *Private* spaces enable confidential conversations, reducing conflict in shared environments (14, 15). *Indoor environmental* factors critically shape residents’ social behaviors (26). Stable temperatures reduce irritability during communal activities, while excessive noise disrupts dialogue. Natural daylight enhances mood and engagement, whereas poor ventilation may fragment social cohesion. Sensory-friendly designs create spaces where trust and conversation thrive (47). Collectively, these elements of the built environment constitute the “environmental press” which, according to the ecological theory of aging, interacts with residents’ personal “competence” to shape their daily social outcomes.

### 2.3 Research gap

Prior work linking the BE to SI typically examines single environmental attributes (e.g., walkability, greenness, or lighting) in community settings and often among mixed-age adults rather than older adults living in care homes (see Supplementary Table 1). Evidence from care homes is comparatively sparse and, where available, tends to address overall quality of life or loneliness rather than the everyday social interactions that sustain wellbeing in congregate living. Moreover, institutional studies frequently rely on self-reports and proxy indicators, provide limited within-facility comparisons, and rarely consider how multiple BE dimensions operate together (e.g., accessibility, functional facilities, and privacy) to shape different forms of SI. As a result, we still lack systematic, setting-specific evidence on how care home environmental configurations influence residents’ SI. This study addresses this gap by focusing on older adults living in care homes, examining multi-dimensional BE features and their associations with observed interpersonal, small-group, and caregiver-related interactions across facilities.

## 3 Conceptual model

**Figure 1:**
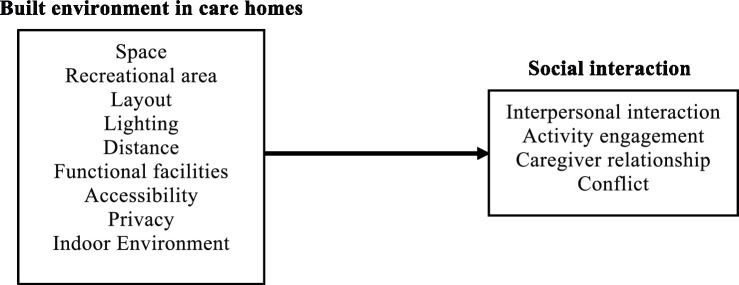
A conceptual BE–SI model for the older adults in care homes.

Based on a comprehensive review of existing literature, it is clear that the built environment plays a pivotal role in influencing SI among older adults. The importance of the BE in shaping social interactions aligns with the ecological theory of aging (48, 49). This study proposes a conceptual model. Nine care-home built-environment (BE) factors are hypothesized to influence social interaction among residents, covering interpersonal interaction, activity engagement, caregiver relationship, and conflict (see Figure 1). This model extends the scope of P–E theory by applying it to care home settings and using it to explain how specific environmental features shape SI among residents. This study underscores the social needs of care home residents and the environment’s potential to support or hinder those needs. By identifying specific environmental features linked to residents’ SI, these findings provide actionable guidance for improving care home environments and, in turn, promoting residents’ mental and physical wellbeing.

## 4 Research methods

### 4.1 Research design

**Figure 2:**
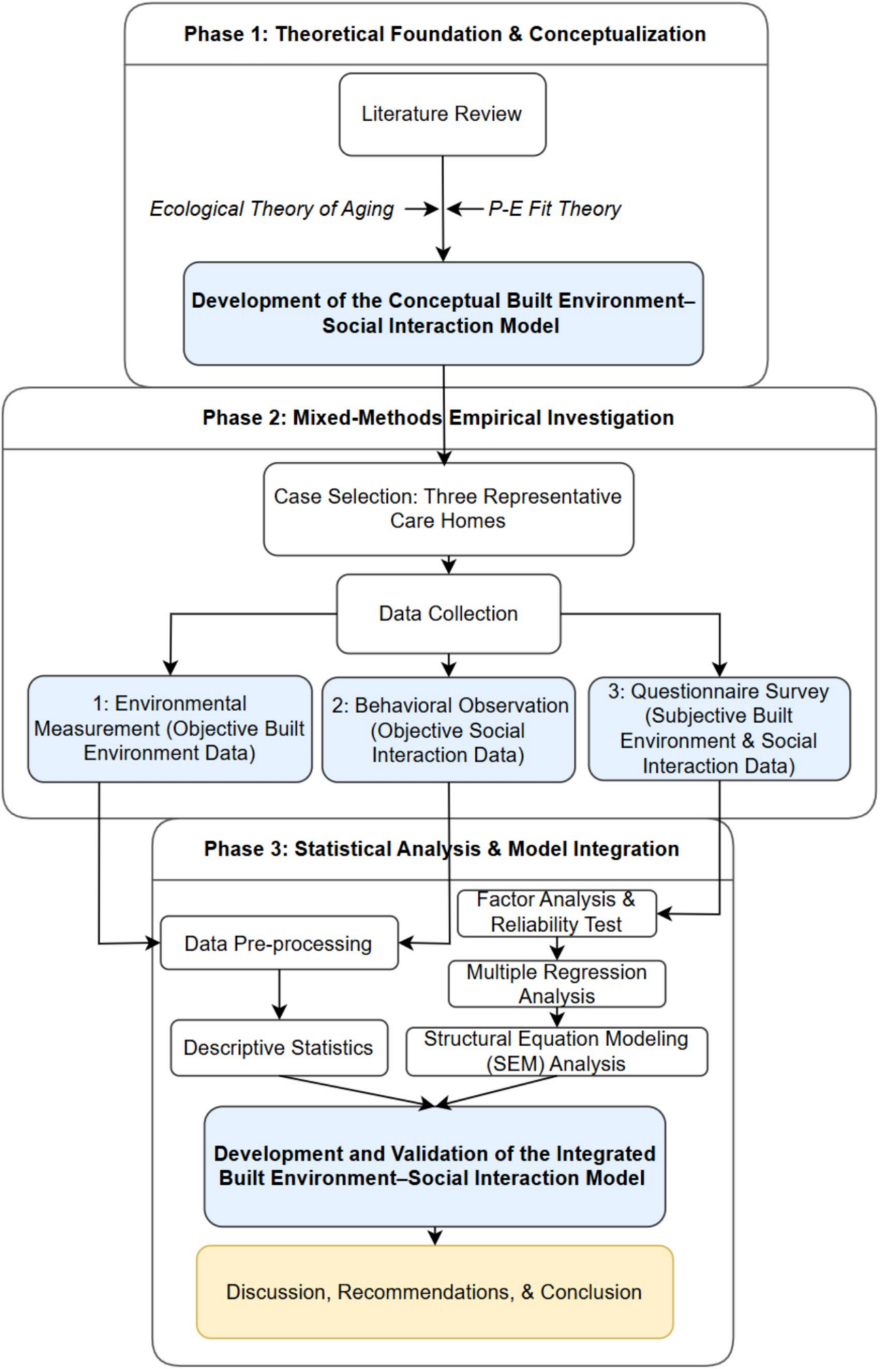
Research framework.

This study employed a mixed-methods research design to systematically investigate the relationships between the built environment (BE) and social interaction (SI) among older adults in care homes. The overall procedural framework of the research is illustrated in Figure 2. The process began with a comprehensive literature review and the establishment of a theoretical foundation based on the ecological theory of aging and p-e fit theory, leading to the development of a conceptual BE-SI model. Subsequently, an empirical investigation was conducted in three care homes, utilizing a concurrent data collection strategy that integrated objective environmental measurements, direct behavioral observations, and a subjective questionnaire survey. Finally, the collected data were subjected to a series of statistical analyses, including factor analysis, multiple regression, and structural equation modeling (SEM), to test the conceptual model and develop a final, integrated BE-SI model.

Three representative care homes were deliberately selected: The first, a large-scale public facility established in 2023, has 12 floors, spans 260,000 square meters, employs 500 staff members, and accommodates 1,000 residents. The two smaller private facilities are Home 2 and Home 3. Home 2, built in 2014, is a four-story building with 2,000 m^2^ of floor area, 20 staff members, and capacity for 60 residents. Home 3, completed in 2020, is a three-story facility with 3,080 m^2^ of floor area, 30 staff members, and capacity for 80 residents. The research procedures were reviewed and approved by the university’s research ethics committee to ensure compliance with ethical guidelines (Ethical Review Number: NJTECH-18; Date: 2024.07.03), and written consent was obtained from all participants through signed consent forms.

### 4.2 Environmental measurement and behavior observation

Indoor environmental measurements were conducted in each care home at approximately 10:00 a.m. on Saturdays using calibrated, high-precision instruments, following international standards. Illuminance was measured with a digital lux meter (TA636A, CEM Instruments, China) per ISO 8995-1:2002, using five points (center and four corners, ≥0.5 m from walls) at 850 mm height to represent seated eye level. The mean of these readings indicated overall lighting conditions. Air temperature and relative humidity were measured at the same five points using a digital thermohygrometer (TA620, CEM Instruments, China), following ISO 7726:1998. Measurements were taken at 1.1 m height, avoiding direct sunlight and ventilation sources. The average values reflected thermal and humidity conditions. Ambient noise was assessed using a Class 1 sound level meter (AR844, SMART SENSOR, China) in line with IEC 61672–1:2013. A-weighted equivalent continuous sound levels (LAeq) were recorded over 2-min intervals at each point, and the mean value was used to characterize the acoustic environment. Between 2:00 p.m. and 4:00 p.m. on the same day, three independent observers conducted structured behavioral observations using a standardized coding form. The final social interaction score was the average of their recorded counts.

### 4.3 Questionnaire survey

Based on the literature review and conceptual model (Figure 1), a questionnaire survey was designed to collect quantitative data from older adults in care homes. The questions were simple and easily understandable, focusing on participants’ subjective perceptions of the BE and their real experiences of social interactions. Given the lack of a single scale that fully integrates both environmental factors and social interactions, items of BE and SI were selected from separate scales that address each dimension independently, while ensuring their relevance to the research objectives. A five-point Likert scale (1 = strongly/extremely disagree, 5 = strongly/extremely agree) was applied to assess participants’ views on SI and BE. The scale had three sections: (1) background information; (2) SI experiences (50, 51); and (3) agreement with the description of BE (26, 39, 52). The survey was administered face-to-face in the activity room. A total of 119 residents took part in the study. These participants met the following inclusion criteria: they were aged 60 years or above, had resided in the care homes for a period exceeding 1 month, and possessed the cognitive and physical ability to understand and respond to the questionnaire items.

### 4.4 Statistical methods

To ensure the reliability and validity of the findings, a range of analytical methods were employed. The quantitative data collected from the questionnaire survey were analyzed using several techniques, including factor analysis, reliability testing, multiple regression analysis, and structural equation modeling (SEM). First, factor analysis was conducted to explore the dimensional structure of the SI items and to group them into distinct factors. Second, reliability tests, specifically using Cronbach’s alpha, were performed to assess the internal consistency of the BE and SI factors. Third, multiple regression analysis was utilized to develop regression models, aiming to identify the relationships between the BE in care homes and the SI of older adults. Lastly, structural equation modeling (SEM) was applied to establish structural models that reveal the relationships between latent and observed variables (53). These comprehensive analyses provided a robust framework for understanding the complex interactions between the BE and SI among older adults in care homes.

## 5 Results

### 5.1 Demographics

This study included 119 participants, with 74% aged 70 years or older (22.7% aged 70–74 years, 24.4% aged 75–79 years, and 26.9% aged 80 years or older). Females comprised 54.6% of the sample, slightly exceeding males (45.4%). Most participants (84.0%) had resided in care homes for at least 3 months, with 37.8% living there for 3 months to 1 year, 31.1% for 1–3 years, and 15.1% for over 3 years. Regarding marital status, 63.0% were widowed, while 37.0% lived with spouses. Educational attainment varied: 55.5% held college degrees or higher, 19.3% completed secondary education, 10.9% had primary education, and 14.3% received no formal education. All participants demonstrated unimpaired verbal communication abilities.

### 5.2 Environment measurement and behavioral observation in care homes

Table 1 compares environmental aspects across three care homes. Homes 1 and 3 have larger residential areas than Home 2. Home 3 features wall holes instead of traditional windows in the dining area. Home 1 has the most extensive recreational area. Regarding equipment, Home 1 contains fitness equipment, Home 3 has cultural items (calligraphy desks, pianos), while Home 2 lacks these amenities. Homes 1 and 3 are better equipped with elevators and call bells than Home 2. Comparative analysis of indoor environments and social interaction (SI) frequency within recreational areas, activity rooms, and dining areas revealed that Home 1 demonstrated higher Total Frequency of SI compared to Homes 2 and 3 (Table 2). The floor plans of the three care homes were briefly mapped out in this study to provide a clearer overview of the spatial structure (see Supplementary Figure 1).

**Table 1:** Detailed information of selected care homes.

**Category**		**Home 1**	**Home 2**	**Home 3**
**Residential area**				
Size (Length × Width × Height m)		10 × 7.5 × 2.8	4 × 3 × 2.5	5.2 × 5.3 × 3
Window	Number	1	1	2
	Size (m)	1.2 × 0.7	0.8 × 1.1	2.8 × 1.5
	Orientation	S	E	E
Number of Elevators		3	/	2
Presence of Call Bells		√	/	√
Type of room		Single room	Single/Double room	Single room
**Recreational area**				
Size (Length × Width × Height m)		300 × 200 × 5	Open space with a plastic shed roof as the covering	12.5 × 8.1 × 3
Window	Number	30	/	5
	Size (m)	2 × 1		1.4 × 1.1
	Orientation	E/S/N/W		E/S
Number of Elevators		6	/	2
Sofa	Number	80	/	4
	Horizontal Spacing (m)	3		0.6
	Vertical Spacing (m)	3		0.6
**Activity room**				
Size (Length × Width × Height m)		25 × 7 × 5	9 × 6 × 2.6	12 × 11 × 2.8
Window	Number	2	2	4
	Size (m)	2 × 1.5	3 × 1.5	1.54 × 0.9
	Orientation	S	E/W	N
Equipment	Type	Treadmill/Stability ball/Dumbbell/Kettlebell	/	Calligraphy desk/Piano/ Treadmill
	Number	10		5
	Spacing (m)	0.8		0.6
**Dining area**				
Size (Length × Width × Height m)		300 × 200 × 5	6 × 2.7 × 2.3	7 × 4.5 × 3.1
Window	Number	30	1	(Holes - making in the wall without windows)
	Size (m)	1 × 0.5	1.3 × 1.2	
	Orientation	E/S/N/W	N	
Dining Table	Number	100	2	4
	Size (m)	2 × 1	1.2 × 0.6	2.2 × 1
	Seats number	4	4	4
**Main connecting roads of different areas**				
Size (Length × Width m)		800 × 4.2	200 × 3	300 × 3
Surface material		Wood	Cement	Compound board
Accessibility		Handrail/Assistive wheelchair/Corner seat	Uneven road surface/Exist steep slope	Handrail/ Corner seat

Building orientation: *N =* North-facing; S = South-facing; E = East-facing; W = West-facing.

**Table 2:** Indoor environment and social interactions of old adults in care homes.

Location	Category		Home 1	Home 2	Home 3
Recreational area	Indoor Environment	Temperature (°C)	**24**	*23*	*23*
		Humidity (%RH)	**76**	**76**	*73*
		Illuminance (lx)	*80*	95	**410**
		Noise (dB)	*60*	63	**66**
	Frequency of social interaction (times)	Individual Interaction	**34**	*18*	28
		Small Group Interaction	**41**	*30*	34
		Group Interaction	**6**	*0*	*0*
		Conflict Behavior	2	**4**	*0*
Activity room	Indoor Environment	Temperature (°C)	**26.6**	21	*20.4*
		Humidity (%RH)	**79.2**	*71*	72.6
		Illuminance (lx)	360	*320*	**380**
		Noise (dB)	**65**	**65**	*60*
	Frequency of social interaction (times)	Individual Interaction	38	*28*	**48**
		Small Group Interaction	**99**	*83*	91
		Group Interaction	*20*	**38**	23
		Conflict Behavior	0	**3**	0
Dining area	Indoor Environment	Temperature (°C)	**25**	**25**	*20.5*
		Humidity (%RH)	79.2	**80**	*72.6*
		Illuminance (lx)	**200**	*180*	190
		Noise (dB)	**66**	*60*	*60*
	Frequency of social interaction (times)	Individual Interaction	**37**	21	*16*
		Small Group Interaction	**60**	16	*12*
		Group Interaction	0	0	0
		Conflict Behavior	*0*	**1**	*0*
Total Frequency of Social Interaction (times)	Recreational area		**83**	*52*	62
	Activity room		157	*152*	**162**
	Dining area		**97**	38	*28*

Bold value: highest value in three homes and Italic value: lowest value in three homes.

### 5.3 Factor analysis and reliability test

Social interaction (SI) factors were identified through factor analysis (Table 3). The sample-to-item ratio was 7:1, exceeding the recommended 5:1 minimum and ensuring robust statistical power (54). The descriptive results (i.e., mean, median, minimum, and maximum scores) for the independent and dependent variables were presented in Supplementary Table 2. All factor loadings were above 0.5, indicating significant item contributions (55). The Kaiser-Meyer-Olkin measure was 0.813, well above the 0.60 threshold, confirming data suitability for factor extraction (56). Internal consistency of both built environment (BE) and SI factors was evaluated using reliability tests (Tables 3, 4). Cronbach’s alpha values exceeded 0.6, considered acceptable (57), indicating agreeable internal consistency. The measures were reliable without requiring further modification, supporting construct validity.

**Table 3:** Factor analysis and reliability of social interaction factors of older people.

Factors	Nature	S/N	IItems	Factor loading	α
*Social interaction* KMO = 0.813					
SI1- Interpersonal interaction	+	SI11	Frequently participate in group conversations	0.901	0.910
	+	SI12	Frequently engage in casual conversations with others	0.874	
	+	SI13	Frequently greet others	0.853	
	+	SI14	Frequently engage in mutual visiting among older residents	0.701	
	+	SI15	Frequently participate in board games or card games	0.660	
	+	SI16	Frequently engage in physical contact with others	0.646	
	+	SI17	Frequently participate in group or communal activities	0.557	
SI2- Activity engagement	+	SI21	Frequently engage in leisure activities such as cooking or watching films	0.810	0.782
	+	SI22	Frequently engage in cultural activities, such as music, chess, calligraphy, or painting	0.780	
	+	SI23	Frequently participate in physical exercise or fitness activities	0.746	
	+	SI24	Frequently engage in outdoor walking activities	0.558	
SI3- Caregiver relationship	+	SI31	Desire to establish positive relationships with staff members	0.853	0.894
	+	SI32	Frequently engage in conversations with staff members	0.852	
	+	SI33	Interact with staff members in a natural and comfortable manner	0.848	
	+	SI34	Maintain a close and friendly relationship with staff members	0.833	
SI4-Conflict	+	SI41	Experience interpersonal conflicts with other older residents	0.912	0.855
	+	SI42	Experience interpersonal conflicts with care staff or community service personnel	0.861	

α = Cronbach’s Alpha Value; KMO = Kaiser-Meyer-Olkin; − The ‘+’ (quotation marks) indicates a positive item and ‘- ‘indicates a negative item.

**Table 4:** Reliability analysis for built environment factors in care homes.

Factors	Nature	S/N	Items	α
Built environment				
BE1-Space	+	BE11	The sun-facing corridors in the residential units are designed to be spacious.	0.890
	+	BE12	The hallways connecting the rooms within the residential areas are adequately wide.	
	+	BE13	The living rooms in the residential areas feature generous spatial dimensions.	
BE2-Recreational area	+	BE21	The entrance space of the recreational area is designed to be spacious.	0.921
	+	BE22	The hallways connecting the rooms within the recreational area are sufficiently wide.	
	+	BE23	The passageways throughout the recreational area provide ample width for circulation.	
	+	BE24	The doorways of the rooms in the recreational area are designed with generous dimensions.	
BE3-Layout	+	BE31	The layout of sofas and tables in the recreational area is thoughtfully arranged for functionality and comfort.	0.890
	+	BE32	The placement of equipment in the activity room is strategically organized to ensure efficient use of space.	
	+	BE33	The overall layout of the care home is appropriately designed.	
BE4-Lighting BE5-Distance	+	BE41	The residential spaces are characterized by good daylighting.	0.837
	+	BE42	The recreational area is well-illuminated by natural light.	
	+	BE43	The distance between the residential area and recreational area is appropriate, ensuring convenient access.	0.852
	+	BE44	The distances between the activity rooms are well-proportioned.	
BE6-Functional facilities	+	BE61	The care home is equipped with a full range of functional rooms and various activity spaces.	0.844
	+	BE62	The rehabilitation equipment in the care home is fully available.	
	+	BE63	The recreational area is equipped with a comprehensive selection of entertainment equipment.	
	+	BE64	The care home is well-equipped with emergency response facilities.	
BE7-Accessibility	+	BE71	The number of elevators in the care home is adequate.	0.830
	+	BE72	The stair design in the care home is appropriate.	
	+	BE73	All passageways are accessible for barrier-free circulation (e.g., with handrails).	
	+	BE74	The activity areas are fully accessible for barrier-free movement.	
BE8-Privacy	+	BE81	The bathrooms and shower rooms offer a high level of privacy.	0.864
	+	BE82	The recreational area provides private spaces for individual communication.	
	+	BE83	Personal belongings in the recreational area are stored with privacy protection.	
BE9-Indoor Environment	+	BE91	The indoor temperature in the recreational area is comfortable.	0.919
	+	BE92	The indoor humidity in the recreational area is maintained at an appropriate level.	
	+	BE93	The noise levels in the recreational area are kept to a minimum.	
	+	BE94	The indoor illumination in the recreational area is adequate.	

α = Cronbach’s Alpha Value; - All items were measured on a 5-point scale, ranging from ‘strongly disagreed’ to ‘agreed’; - The ‘+’ (quotation marks) indicate statements that contained a positive meaning in relation to the factor.

### 5.4 Multiple regression analysis

The multiple regression analysis was used to develop four models to examine the relationships between BE factors and SI among older adults (see Table 5). Model 1 indicated that functional facilities (BE6) positively influenced interpersonal interaction (SI1), while privacy (BE8) had a negative effect. This model explained 7.9% of the variance in SI. Model 2 demonstrated that accessibility (BE7) was a significant positive predictor of activity engagement (SI2), accounting for 16.0% of the variance. Model 3 showed that accessibility (BE7) also positively affected caregiver relationships (SI3), explaining 8.3% of the variance. Finally, Model 4 found that recreational areas (BE2) and lighting (BE4) negatively impacted conflict (SI4), with the model accounting for 21.1% of the variance. These models collectively highlighted the critical role of built environment factors in shaping SI, explaining between 7.9 and 21.1% of the variance in SI outcomes.

**Table 5:** Multiple regression model for built environment factors and social interaction of older people.

Models		B	S. E.	Sig.	95% CI	q (FDR)	VIF	R	R^2^	ANOVA	
										F	Sig.
1	Interpersonal interaction	←				BE Factors					
	Constant	−0.478	0.514	0.355				0.281	0.079	4.975	0.008
	BE6 Functional facilities	0.328	0.118	0.006	[0.097, 0.559]	0.012	1.151				
	BE8 Privacy	−0.224	0.093	0.018	[−0.406, −0.042]	0.018	1.151				
2	Activity engagement	←				BE Factors					
	Constant	−1.749	0.380	0.000				0.400	0.160	22.228	0.000
	BE7 Accessibility	0.428	0.091	0.000	[0.250, 0.606]	q < 0.001	1.000				
3	Caregiver relationship	←				BE Factors					
	Constant	−1.265	0.397	0.002				0.289	0.083	10.659	0.001
	BE7 Accessibility	0.310	0.095	0.001	[0.124, 0.496]	q = 0.001	1.000				
4	Conflict	←				BE Factors					
	Constant	3.210	0.585	0.000				0.459	0.211	15.469	0.000
	BE2 Recreational area	−0.406	0.150	0.008	[−0.700, −0.112]	0.016	1.657				
	BE4 Lighting	−0.313	0.151	0.041	[−0.609, −0.017]	0.041	1.657				

S. E. = standard error; Sig. = significance; VIF = variance inflation factor. Multiplicity and effect sizes. For each model, p-values are accompanied by Benjamini–Hochberg FDR–adjusted q-values. Effect sizes are shown as unstandardized coefficients with 95% confidence intervals. Coefficient–significance matrix of all predictor was shown in Supplementary Table 3.

Regression assumptions were checked in SPSS (see Supplementary Figures 2a–2d). Linearity was assessed by inspecting standardized-residuals versus predicted values; plots showed approximately random scatter with no systematic curvature across models. The histogram and Normal P–P plots indicated acceptable normality for Model 3 (Shapiro–Wilk *p* = 0.192), with departures for Models 1, 2, and 4 (Shapiro–Wilk *p* = 0.005, 0.045, and < 0.001, respectively). Homoscedasticity was examined via residual plots and a Glejser test; Models 1–3 showed no evidence of heteroskedasticity (*p* = 0.206, 0.323, 0.133), whereas Model 4 indicated heteroskedasticity (*p <* 0.001). All variance inflation factors were below 2 (Max VIFs = 1.151, 1.000, 1.000, 1.657), indicating no multicollinearity. These diagnostics suggest that the multiple linear regression assumptions were adequately met for Models 1–3 and partially met for Model 4; deviations are noted and results are interpreted with appropriate caution (see Supplementary Table 5).

### 5.5 Structural equation model

**Figure 3:**
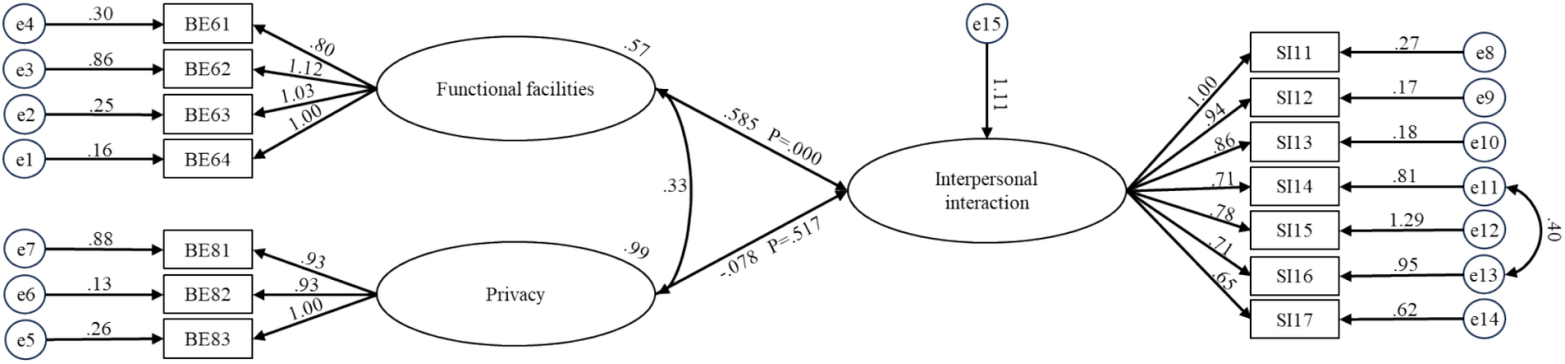
Structural equation model for BE-interpersonal interaction for older people in care homes.

**Figure 4:**
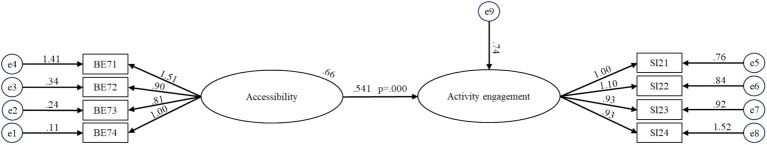
Structural equation model for BE-activity engagement for older people in care homes.

**Figure 5:**
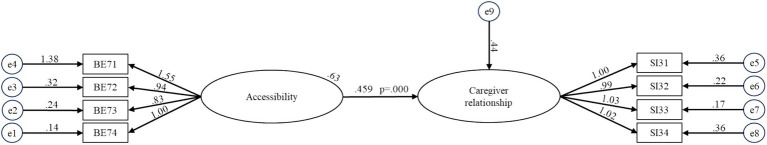
Structural equation model for BE-caregiver relationship for older people in care homes.

**Figure 6:**
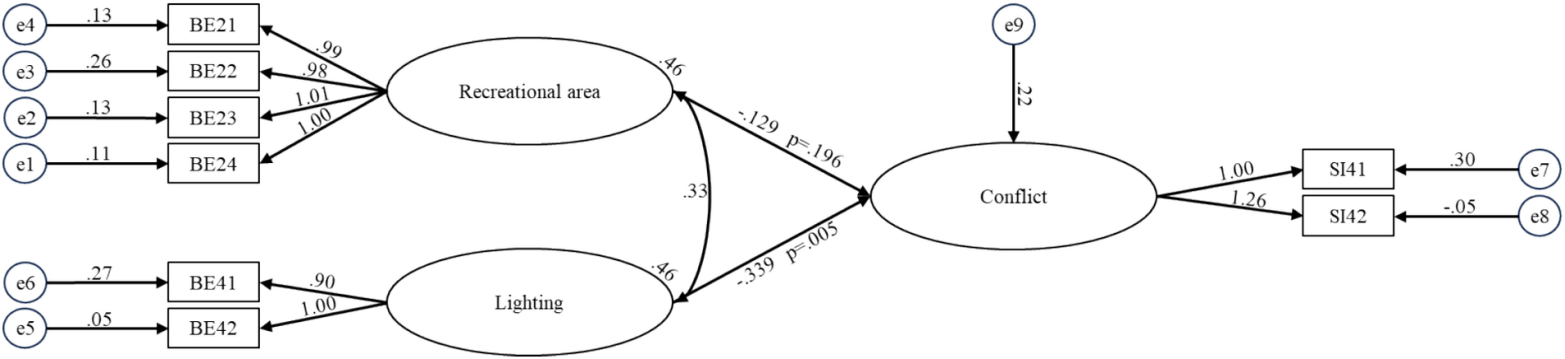
Structural equation model for BE-conflict for older people in care homes.

Structural equation models (SEMs) were developed based on the multiple regression models presented in Figures 3–6. The model fit was considered acceptable when the following criteria were met: x^2^/df < 3, RMSEA < 0.08, GFI > 0.90, CFI > 0.90, IFI > 0.90, and TLI > 0.90 (58, 59). The values of sample-to-parameter ratio were ranged from 7:1 to 12:1, satisfying the lowest requirement of 5:1 (60). In this study, the SEMs were deemed satisfactory as at least four fit indices met recommended thresholds (61, 62). Hence, although some of the indices was not met the acceptable value (e.g., RMSEA), the model is acceptable because at least four other fit indices met recommended thresholds (79, 80). The model labeled BE-Interpersonal Interaction I was constructed based on the relationships between built environment factors and interpersonal interaction (SI1), as identified in the regression analysis (see Figure 3). To enhance model fit, an additional path was incorporated between “Frequently engage in mutual visiting among older residents” (SI14) and “Frequently engage in physical contact with others” (SI16), resulting in the revised BE-Interpersonal Interaction II model (x^2^/df = 2.532, RMSEA = 0.114, CFI = 0.903, IFI = 0.905, TLI = 0.901).

The BE-Activity Engagement model was developed to illustrate the influence of the BE on activity engagement (SI2) among older residents in care homes (see Figure 4). The model demonstrated an adequate fit with the following indices: x^2^/df = 2.533, RMSEA = 0.114, GFI = 0.906, CFI = 0.932, and IFI = 0.933. To assess the relationship between the BE and caregiver relationships (SI3) of older adults, the BE-Caregiver Relationship model was formulated, which also exhibited an acceptable fit (x^2^/df = 2.054, RMSEA = 0.095, GFI = 0.928, CFI = 0.965, IFI = 0.965) (see Figure 5). Finally, the BE-Conflict model was constructed to capture the interactions between the BE and conflict (SI4) among older residents (see Figure 6). This model exhibited excellent fit, with indices of x^2^/df = 1.212, RMSEA = 0.042, GFI = 0.959, CFI = 0.994, and IFI = 0.995.

The maximum likelihood estimates for the four optimal structural equation models are presented in Figures 3-6. The results reveal the following findings: older residents’ agreement with functional facilities was found to have a positive effect on interpersonal interaction, with a standardized path coefficient of 0.585, and this relationship was statistically significant (*p <* 0.001) (BE6-SI1). In contrast, privacy showed a negative path coefficient of −0.078, but this relationship was not statistically significant (*p* = 0.517), indicating no meaningful impact on interpersonal interaction (BE8-SI1). Accessibility was positively associated with both activity engagement and caregiver relationships, with standardized path coefficients of 0.541 (*p <* 0.001) for activity engagement (BE7-SI2) and 0.459 (*p <* 0.001) for caregiver relationships (BE7-SI3). The availability of recreational areas showed an inverse relationship with conflict, with a path coefficient of −0.129, but this effect was not statistically significant (*p* = 0.196) (BE2-SI4). Lastly, lighting had a negative path coefficient of −0.339, which was statistically significant (*p* = 0.005), indicating a substantial effect on conflict (BE4-SI4). SEM structural paths with effect sizes (95% CIs) and FDR-adjusted q-values were presented in Supplementary Table 4.

### 5.6 Model establishment

**Figure 7:**
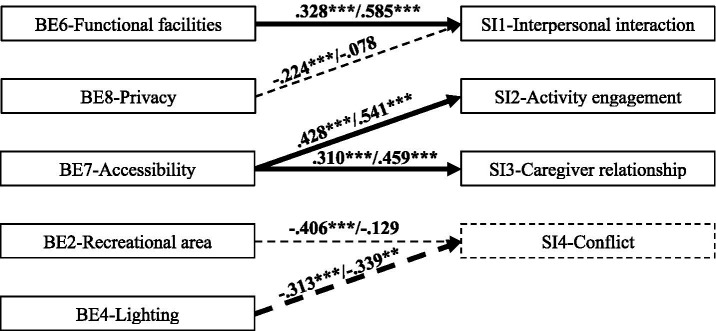
An integrated BE–SI model for older people in care homes. 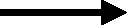 a positive relationship confirmed by regression and SEM; 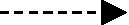 a negative relationship confirmed by regression; 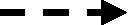 a negative relationship confirmed by regression and SEM. 
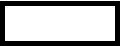
 positive factors; 
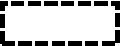
 negative factors.

Through the construction of regression models and structural equation models (SEM), an integrated BE–SI model was developed (see Figure 7). This BE–SI model illustrates the relationships between the BE in care homes and SI among older adults. The final model demonstrates that the richness of functional facilities has a facilitating effect on interpersonal interaction, a relationship supported by both the regression model and SEM. On the other hand, an excessive emphasis on privacy appears to hinder interpersonal interaction, suggesting that an overemphasis on private space can limit opportunities for SI. Furthermore, the regression equation and SEM both indicate that good accessibility significantly enhances activity engagement and caregiver relationship. Additionally, the insufficient configuration of recreational areas and inadequate lighting can exacerbate conflicts.

## 6 Discussion

The study’s findings can be effectively interpreted through the lens of the ecological theory of aging, which posits that social outcomes are a function of the fit between residents’ competence and their environmental press (13). Specifically, functional facilities and privacy were found to significantly influence interpersonal interactions. Homes 1 and 3, equipped with diverse facilities such as treadmills, dumbbells, calligraphy tables, and open communal areas, recorded 38 and 48 instances of individual interactions, respectively, during a two-hour observation in activity rooms. In contrast, Home 2, lacking similar facilities, recorded only 28 instances (Table 2). Functional facilities not only promote group participation but also facilitate spontaneous exchanges during unstructured moments, such as waiting or resting in shared spaces. This pattern confirms prior evidence that amenity-rich environments are associated with greater informal contact and participation among older adults, whereas amenity-poor settings dampen spontaneous exchanges during unstructured time (63, 64). Privacy presents a more nuanced effect. While essential for residents’ sense of security and respect, excessive privacy can restrict social opportunities (14, 15). Home 2 is unique in offering double rooms, which provide lower privacy than the single rooms in Homes 1 and 3. Shared bathrooms in Home 2 may lead to privacy concerns; however, residents there engage more frequently in casual interactions and room visits. Conversely, the higher privacy of single rooms in Homes 1 and 3 limits social interactions within personal spaces, which aligns with evidence that privacy gains may trade off with fewer incidental contacts in residential care settings (63), highlighting a trade-off between privacy and social engagement in care home design.

*Activity engagement* depends heavily on *accessibility*. Observational data indicates that Home 1, with its six elevators and wide corridors (4.2 meters), recorded 41 instances of small group interaction during the observation period, compared to only 30 in Home 2, which lacks elevators and has narrower corridors (Table 2). When the environmental press in the form of physical barriers exceeds residents’ physical competence, participation is suppressed. Conversely, proper accessibility ensures a better person-environment fit, enabling residents to independently navigate between spaces and participate in activities without relying on assistance (65). This interpretation is consistent with studies showing that corridor width, circulation, and overall spatial configuration influence encounter rates and engagement in long-term care environments (63, 66). Wide corridors and barrier-free pathways, as seen in Home 1 and Home 3, allow seamless movement between different areas, fostering greater participation in both structured activities, such as group exercises, and unstructured activities, such as walking or painting. As shown in Table 1, the accessible pathway in Home 2 has an inappropriate slope and insufficient reserved space, significantly reducing the accessibility of Home 2. This increases the difficulty for older residents to reach social areas, which is reflected in the observed lower frequency of social interactions in these areas compared to the other two care homes.

The *caregiver relationship* dimension reflects the frequency and quality of interactions between residents and care staff. *Accessibility* emerged as a significant factor influencing these interactions. Observational data suggests that the accessible design of Home 1 and Home 3 reduces the environmental press on both residents and staff, providing ample and suitable space for interactions. This improved person-environment fit fosters a sense of trust and comfort, encouraging residents to engage with caregivers more frequently. For example, caregivers in Home 3 were observed moving easily between activity rooms, recreational areas, and dining spaces, increasing their visibility and availability to residents. This design fosters a sense of trust and comfort, encouraging residents to engage with caregivers more frequently. Additionally, the design of communal spaces plays an indirect but important role in caregiver relationships (65). This is consistent with studies showing that improved circulation and proximity—key aspects of accessibility—are associated with more frequent, low-effort staff communication and contact (66). Recreational areas in Home 1 are equipped with comfortable seating, large windows providing natural light, and open layouts, creating inviting environments where residents and caregivers can interact. These spaces enable staff to observe and support residents during activities, providing assistance when needed and engaging in casual conversations. In contrast, Home 2’s limited recreational area and restricted accessibility reduced opportunities for such interactions, likely contributing to weaker caregiver-resident bonds.

Conflict behavior is significantly negatively influenced by the recreational area and lighting. The size and layout of the recreational area directly influence the potential for conflicts. In Home 1, the ample space lowers the negative environmental press associated with crowding, allowing residents to participate in activities without encroaching on others’ personal space and thus reducing friction (see Table 1). By comparison, the constrained layout in Home 2 likely exacerbates tensions during group activities, as residents may feel overcrowded or frustrated by limited resources. With increasing light levels in care homes, conflicts in indoor spaces tend to decrease (see Table 2). This effect can be attributed to brighter lighting in recreational areas, activity rooms, and dining spaces, which creates a brighter and more visually comfortable environment. Such conditions contribute to the cultivation of positive emotions and the reduction of stress, ultimately leading to a decrease in conflict occurrences (67). Conversely, the dim lighting and narrow space in Home 2 create an excessive environmental press that can overwhelm residents’ coping competence, contributing to feelings of confinement or irritability and increasing the likelihood of interpersonal conflict.

## 7 Practical recommendations

Recreational areas are the social heart of a care home, and their design should go beyond simply providing space; it must actively cultivate a harmonious social ecosystem. As this study found that inadequate recreational areas can exacerbate conflict, the design should offer a spectrum of social opportunities. This means creating not just large, open zones for group activities, but also semi-enclosed nooks for quiet one-on-one conversations and designated areas for small group pastimes like card games. This design implication is contextually original yet consistent with prior work: studies recommend small, semi-enclosed alcoves linked to public spaces to vitalise everyday encounters in care homes (63), and research on small-scale/household settings reports benefits of dispersed, small social areas over a single large common room (68, 69). By providing this variety, residents can choose their desired level of engagement, reducing the friction that arises from competing social needs in a single, monolithic space. Furniture should then be arranged to support these diverse “social niches,” facilitating everything from intimate chats to lively group discussions.

This study’s finding that poor *lighting* correlates with increased conflict suggests that lighting design is not merely a technical issue of visibility, but a critical tool for regulating the emotional atmosphere of a space. The primary goal should be to create a bright, open, and non-threatening environment that promotes positive emotions and psychological security. Maximizing natural light is paramount, as it connects residents to the outdoors and daily rhythms. For artificial lighting, beyond meeting illuminance standards (300–500 lx), the focus should be on creating a gentle, warm ambiance that is psychologically calming (70). By treating light as a key factor in emotional wellbeing, we can design spaces that proactively de-escalate tension and foster more positive social interactions.

As this research demonstrates a direct link between *functional facilities* and increased interpersonal interaction, their provision should be viewed as an investment in the community’s social capital. Beyond simply offering a diverse range of equipment, the strategic placement of these facilities is key to transforming them into social hubs. For instance, placing comfortable seating near rehabilitation equipment or a tea station next to a calligraphy table encourages the “spontaneous exchanges during unstructured moments” observed in this study. The goal is to design for the social life that happens around the activity, not just the activity itself. This turns functional spaces into vibrant points of connection, sparking conversations and shared experiences.

*Accessibility* is arguably the most critical foundation for a thriving social environment, as our findings link it strongly to both activity engagement and positive caregiver relationships. The design philosophy should extend beyond mere compliance with codes; it should aim to maximize resident autonomy and dignity (71). When a resident can independently and safely navigate from their room to a recreational area, they are empowered to choose to be social. This sense of independence reduces reliance on staff for basic mobility, transforming caregiver interactions from logistical tasks into more meaningful social exchanges. Therefore, wide corridors, intuitive layouts, and ample elevators are not just conveniences; they are instruments of empowerment that grant residents control over their own social lives.

The finding that excessive privacy can negatively correlate with interpersonal interaction calls for a nuanced design approach that balances personal sanctuary with social invitation (26). Instead of “reducing privacy,” the goal should be to create a gradient of spaces. This includes the fully private resident’s room, but critically, also involves designing inviting semi-private or semi-public transitional zones—such as a small seating area outside a cluster of rooms, or a bay window with a bench in a wide corridor. These spaces act as a low-pressure “front porch,” allowing residents to observe the daily flow of life and engage in casual interactions without committing to the full social demands of a large common area. They gently lure residents out of isolation and into the life of the community.

## 8 Limitations and future study

This study intentionally focused on BE determinants of SI among residents rather than including demographic or personality variables. Under the Person–Environment (P–E) fit framework, previous research has emphasized that the individual (P) and environmental (E) components can be examined independently to clarify their respective mechanisms (13, 72). This study aimed to identify the environmental effects, how nine BE factors of care homes influence older residents’ SI, rather than to construct a full predictive model of social behavior. Moreover, prior evidence suggests that accessibility, facilities and layout are key drivers of social interaction in care environments (34, 35). Therefore, focusing solely on BE variables is theoretically justified and consistent with the study’s exploratory objective of isolating environmental contributions within the P–E theoretical framework. Future studies could incorporate key demographic and person-related variables to more rigorously elucidate the underlying mechanisms.

Although the R^2^ values of the regression models (7.9 and 8.3%) appear modest, such levels are common and theoretically acceptable in social and environmental psychology research (73), where human behavior is influenced by multiple interacting factors. Small-to-medium effect sizes can still carry substantive meaning in behavioral research, especially when the goal is to illuminate underlying mechanisms rather than to maximize predictive accuracy (74, 75). In the context of residential care settings, even a small proportion of explained variance suggests that environmental attributes make a measurable and practically relevant contribution to residents’ SI, which are otherwise shaped by diverse personal and contextual variables. Moreover, the relatively homogeneous demographic characteristics of institutional residents may further constrain the variance of SI, making modest R^2^ values theoretically reasonable. Future work should incorporate key demographic and person-related variables (e.g., age, sex, cognitive/functional status, length of stay) to account for residual variance in social interactions and thereby enhance model precision and inferential accuracy.

In the study, lighting, thermal, and acoustic conditions were recorded once at 10:00 on a Saturday using standard instruments, yielding a snapshot rather than a diurnal or weekly average. Because illumination (daylight), temperature/humidity (HVAC cycles and outdoor weather), and sound levels (activity schedules) fluctuate across times of day and days of the week, the estimates reflect associations at that measurement time rather than conditions throughout the facilities. To address this limitation, future studies should use repeated measurements across morning/midday/evening and weekday/weekend periods—or continuous data logging—and align time-stamped environmental data with behavioral observations to better capture temporal variability and reduce measurement error.

## 9 Conclusion

To promote SI among older adults in care homes, the BE must be designed carefully. All of these aspects play a critical role in supporting their SI needs. These needs encompass interpersonal interaction, activity engagement, caregiver relationships, and conflict resolution. It is essential to explore the interactions between the built environment and interpersonal interactions among older adults. This study investigates the effects of older individuals’ subjective perceptions of the BE on their social interactions. Besides questionnaire surveys among 119 older residents in three care homes, environmental measurement and behavioral observation were also conducted. An integrated BE-SI model was developed, utilizing a combination of multiple statistical methods. The final model reveals that: (1) Functional facilities positively influence interpersonal interaction, highlighting the importance of diverse and well-equipped spaces to foster engagement; (2) privacy shows a slight negative correlation with interpersonal interaction, suggesting that while privacy is essential, excessive isolation may hinder social connections; (3) Accessibility has a strong positive impact on both activity engagement and caregiver relationships, emphasizing the need for barrier-free designs; and (4) recreational areas and lighting conditions reduce the conflicts in care homes.

To foster an age-friendly environment for older adults in care homes, several key design elements are essential. Optimizing accessibility in care home design should be a primary consideration for promoting SI among older adults. The proposed measures, such as including 3-meter-wide corridors to ensure unobstructed mobility between key functional zones, strategically positioned elevators to reduce spatial exclusion, and cognitively accessible signage to empower navigation independence, collectively address both physical and perceptual barriers. This study provides valuable insights for designers, facility managers, and staff members in care homes. Furthermore, it contributes to the ecological theory of aging by empirically identifying specific built environment factors—such as functional facilities, accessibility, and lighting—that constitute critical forms of “environmental press” in modern care settings. The developed BE-SI model serves as a practical application of the P-E fit concept, offering a more nuanced framework for creating supportive environments. This enables a better understanding of the critical needs of older adults. This study offers practical guidance on how to effectively design and manage the built environment in these settings to better support the wellbeing and interpersonal interactions of older residents. This study offers insights into BE impact on SI in care homes, but despite incorporating environmental data and behavioral observations, it remains limited by self-report bias (76). Future research should adopt longitudinal designs and integrate electroencephalogram (EEG), virtual reality (VR), and machine learning for more precise analysis (77).

## Data Availability

The raw data supporting the conclusions of this article will be made available by the authors, without undue reservation.
