# Supplementary figures and images for: A mixed-methods investigation for effects of built environments on older people’s social interaction in care homes
Source: Front Public Health. 2025 Oct 29;13:1693935. doi: 10.3389/fpubh.2025.1693935 (PMC12605021; doi:10.3389/fpubh.2025.1693935)

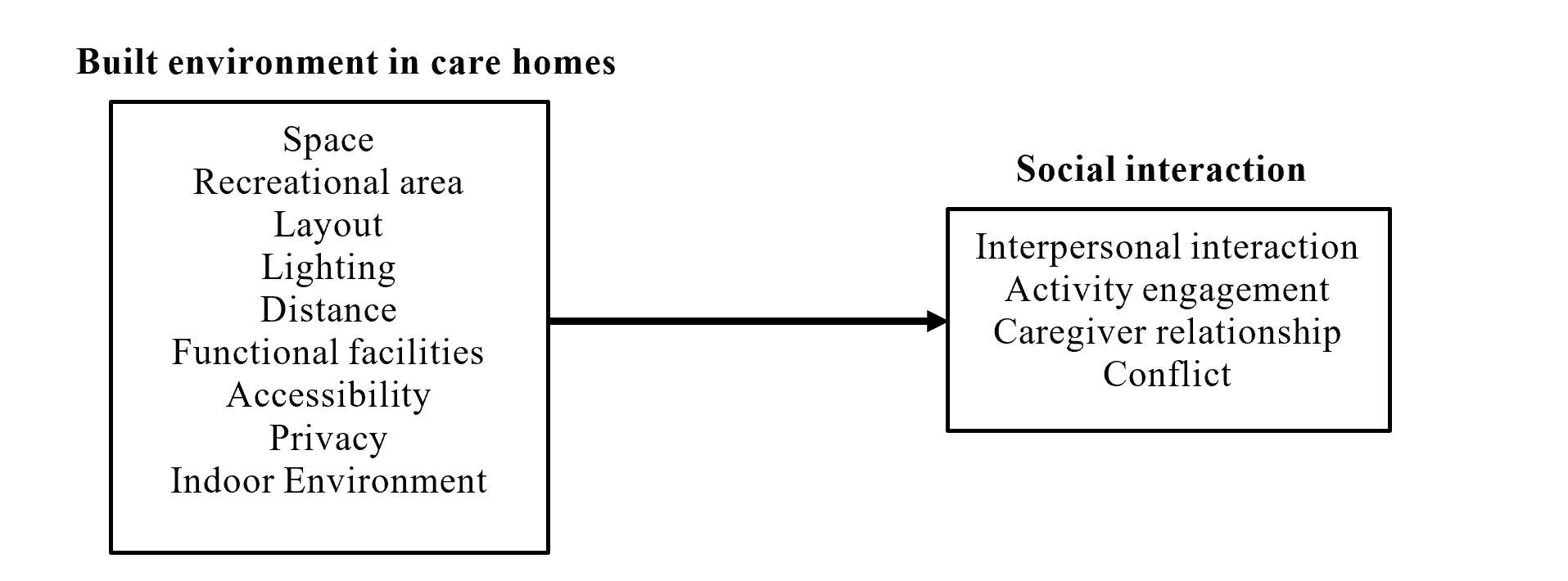

Supplement: Supplementary file 1 [file Data_Sheet_1.zip › figure 1.png]

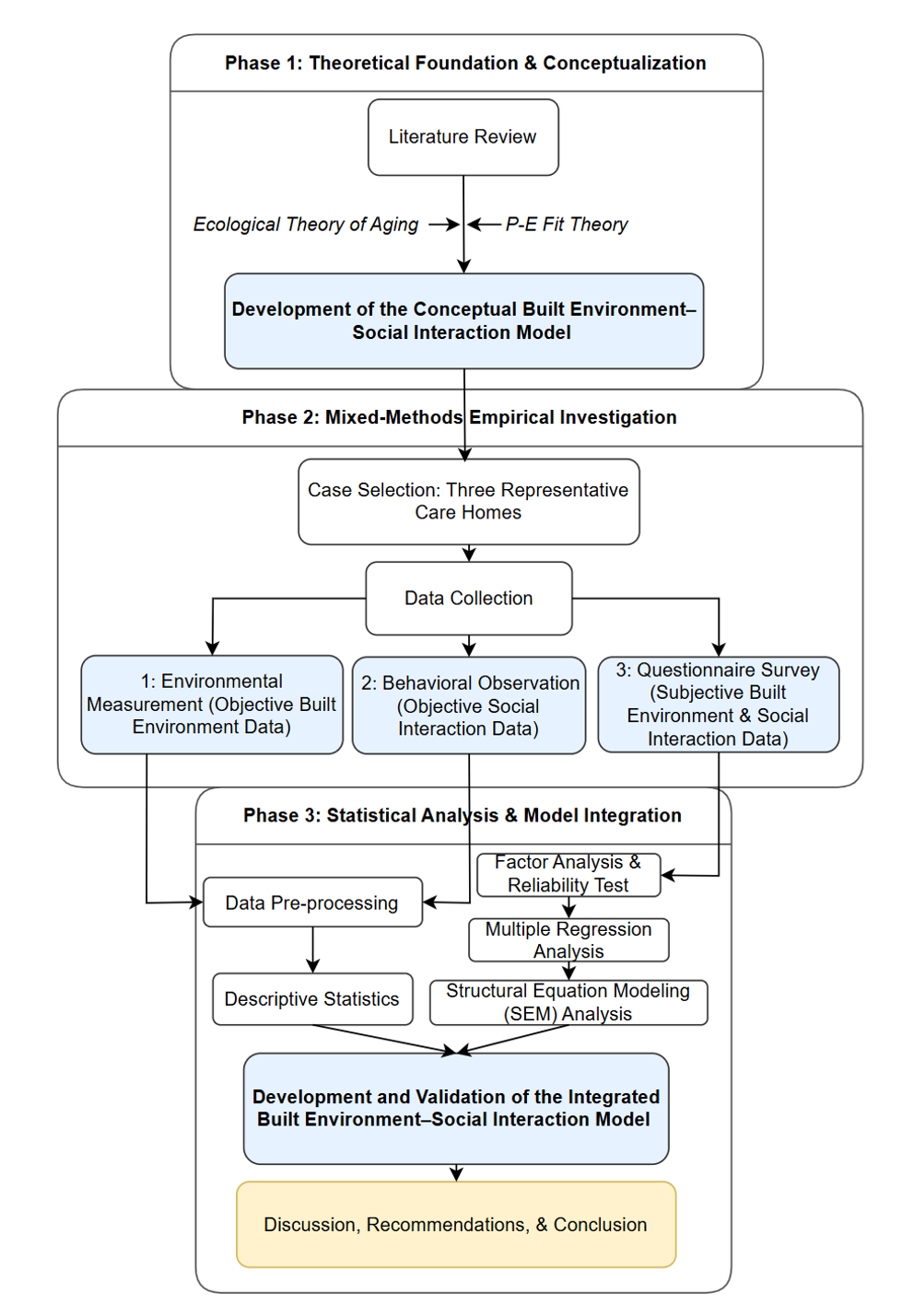

Supplement: Supplementary file 1 [file Data_Sheet_1.zip › figure 2.png]

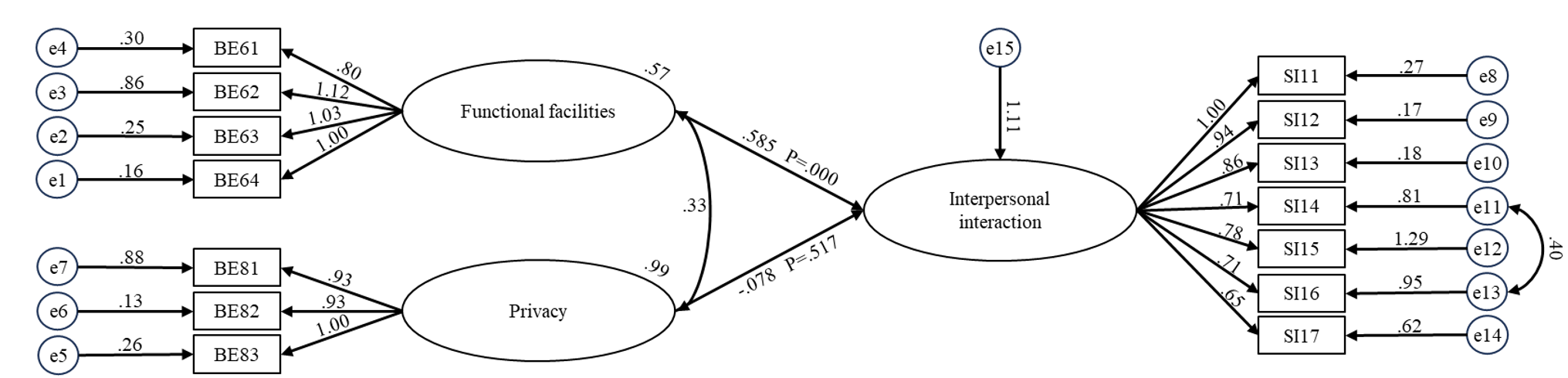

Supplement: Supplementary file 1 [file Data_Sheet_1.zip › figure 2a.png]

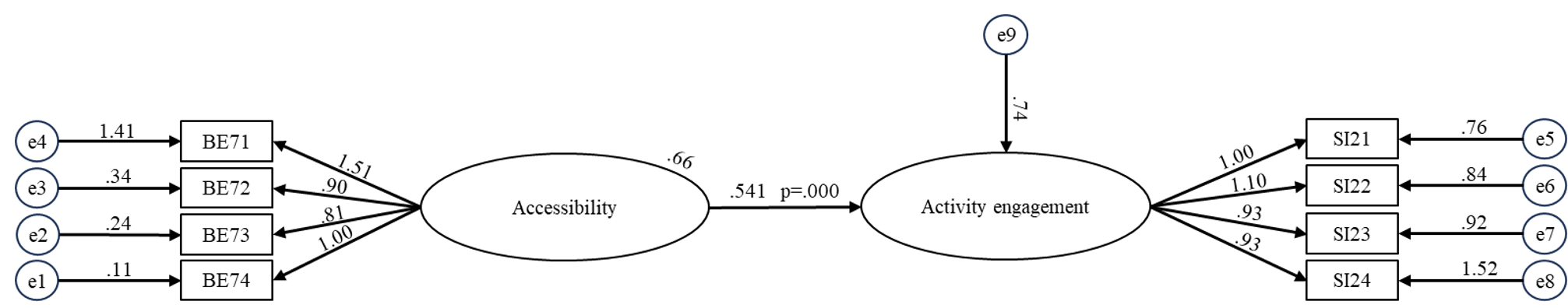

Supplement: Supplementary file 1 [file Data_Sheet_1.zip › figure 2b.png]

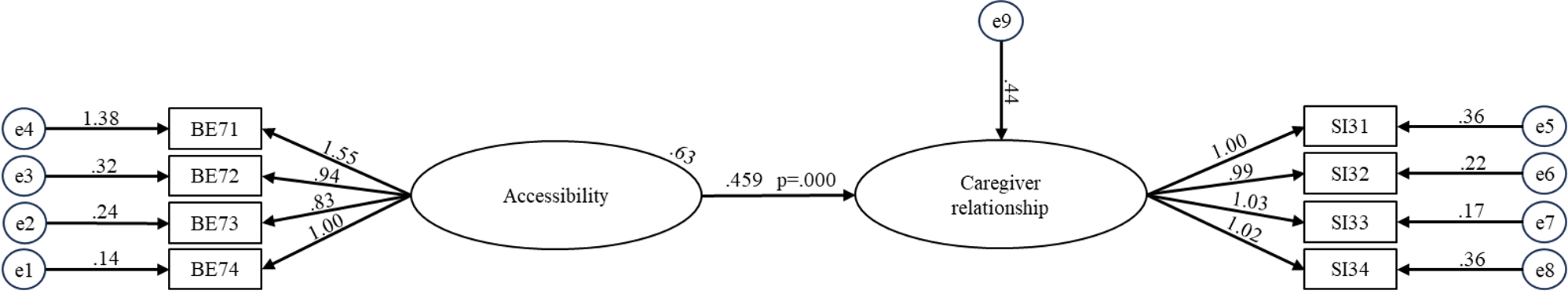

Supplement: Supplementary file 1 [file Data_Sheet_1.zip › figure 2c.png]

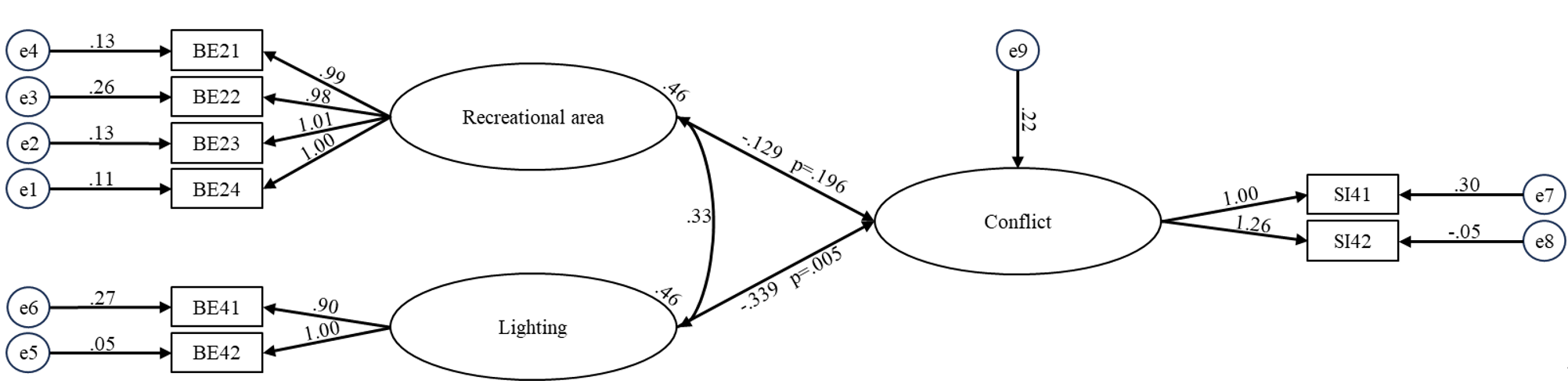

Supplement: Supplementary file 1 [file Data_Sheet_1.zip › figure 2d.png]

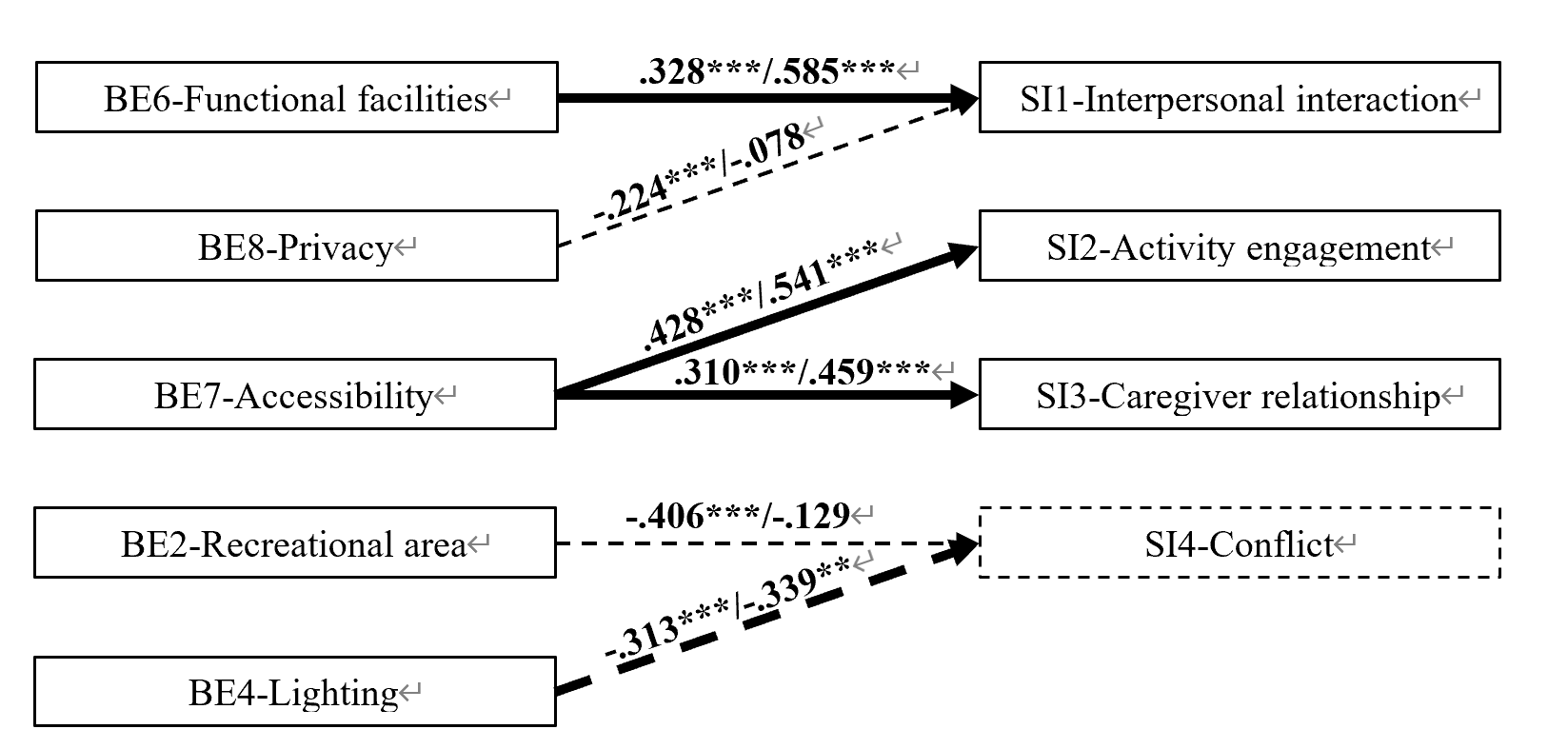

Supplement: Supplementary file 1 [file Data_Sheet_1.zip › figure 3.png]
