# Supplementary material for: A mixed-methods investigation for effects of built environments on older people’s social interaction in care homes
Source: Front Public Health. 2025 Oct 29;13:1693935. doi: 10.3389/fpubh.2025.1693935 (PMC12605021; doi:10.3389/fpubh.2025.1693935)
Supplement: Supplementary file 2 [file Supplementary_file_1.docx]

**Table l Studies about built environment and social interaction**

| **Year** | **Population** | **Factors** | **Methods** | **Results** | **Reference** |
| --- | --- | --- | --- | --- | --- |
| 2024 | Older adults | BE: Accessibility, spatial connectivity, third places, corridor width, seating  SI: Social engagement, co-presence, informal interaction. | Systematized multi-database search; 55 studies screened; inductive thematic analysis. | Accessible circulation and proximate semi-open communal spaces increase co-presence and spontaneous interactions | Gripko & Joseph, 2024 |
| 2024 | 45 residents in a mid-sized Canadian city | BE: Sidewalk continuity/width, visibility.  SI: On-route social interactions (greetings, chats with “familiar strangers”), belonging. | Walking diaries, maps, semi-structured interviews; thematic coding. | Visible BE modifications (e.g., traffic calming, pop-up seating) increased incidental interactions and sense of community | Kuzuoglu *et al*., 2024 |
| 2021 | Workers in workplace | BE: Spatial configuration metrics (integration, choice, connectivity, visibility)  SI: Encounters/co-presence as precursors to interaction. | Theory and accumulated empirical applications | Higher integration/visibility networks reliably increase movement and encounter rates, boosting opportunities for unplanned contact; provides a formal mechanism to link layout and interaction. | Sailer & Koutsolampros, 2021 |
| 2004 | Person in 38 UK residential/nursing homes | BE: Circulation, day spaces, private rooms, bathrooms, outdoor access  SI: QoL domains including social life/relationships; staff morale. | Environmental audit (SCEAM) and resident QoL including proxies/observation; model comparisons via likelihood (Δχ²) per domain. | Community, choice/control, personalization, comfort domains positively associated with QoL; over-emphasis on “safety & health” correlated with lower QoL among less-dependent residents | Parker *et al*., 2004 |
| 2023 | Adults over 18 years old | BE: Walkability, public/green/blue space, transit, housing design, accessibility, third places.  SI: Loneliness / social isolation. | PROSPERO-registered search; eligibility with explicit BE exposure; risk-of-bias appraisal; grouped synthesis. | Walkability/greenspace/third-places frequently linked to lower loneliness | Bower *et al*., 2023 |
| 2023 | Residents in Care & Attention homes | BE: Lighting  SI: QoL social relationships dimension | Correlation/Regression and SEM to build an integrated model linking LE to QoL via mediators (sleep/mood). | Better Lighting (uniformity/daylight in bedrooms, corridor/dining luminance) associated with higher QoL, including social-relationship subdomain; common shortfall was poor lighting control, flagged as an actionable facility target. | Leung *et al*., 2023 |
| 2022 | Older adults | BE: AFC physical environment (facilities, mobility/access, public spaces)  SI: QoL social resources | Reliability analysis, correlation analysis and structural equation modeling (SEM) | Environmental QoL and social QoL were both influenced by outdoor spaces, communication and information and community and health services. | Yu *et al*., 2022 |
| 2003 | City residents | BE: Walkability  SI: Social capital (knowing neighbours, trust, political/associational participation) | Household survey; multivariable analyses comparing neighbourhood types. | Residents in walkable, mixed-use areas reported higher social capital (more neighbour ties, trust and participation) | Leyden, 2003 |
| 2019 | Community-dwelling older adults. | BE: Sidewalk condition, facilities for daily life & exercise, natural environment, design-related safety  SI: QoL including social-relationships domain. | Multiple Linear Regression and SEM to estimate direct effects by QoL domain. | Perceptions of neighborhood environment have diverse impacts on their QoL | Zhang & Li, 2019 |


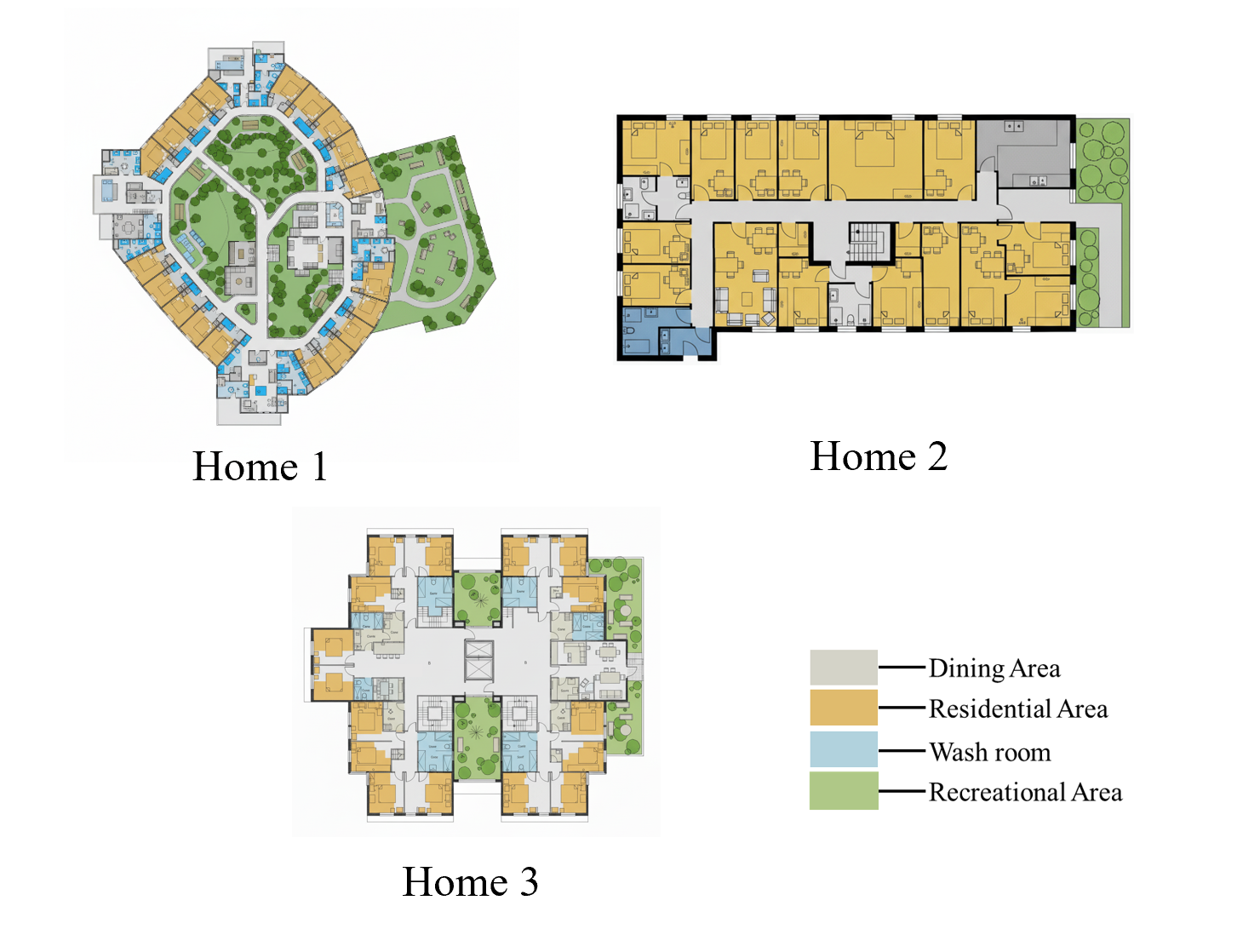


Figure 1 Floor plan of three care homes

Table 2 Descriptive statistics of three care homes

| **Factors** | **Home 1** | | | | **Home 2** | | | | **Home 3** | | | |
| --- | --- | --- | --- | --- | --- | --- | --- | --- | --- | --- | --- | --- |
|  | Med | Mean | Max | Min | Med | Mean | Max | Min | Med | Mean | Max | Min |
| **Social Interaction** | | | | | | | | | | | | |
| Interpersonal interaction | 0.32 | 0.18 | 1.71 | -1.67 | -0.01 | 0.03 | 1.57 | -2.02 | 0.09 | -0.17 | 1.4 | -2.6 |
| Activity engagement | 0.07 | 0.1 | 1.22 | -2.08 | -0.39 | -0.4 | 1.64 | -2.9 | 0.4 | 0.32 | 1.79 | -1.97 |
| Caregiver relationship | 0.69 | 0.64 | 2.09 | -1.6 | -0.25 | -0.34 | 1.55 | -2.37 | -0.01 | -0.15 | 1.81 | -2.44 |
| Conflict | -0.61 | -0.54 | 0.54 | -1.17 | -0.02 | 0.35 | 3.72 | -1.01 | -0.3 | 0.06 | 2.65 | -0.83 |
| **Bulit Environment** | | | | | | | | | | | | |
| Space | 5 | 4.81 | 5 | 4 | 3.67 | 3.83 | 5 | 2.67 | 5 | 4.71 | 5 | 3.67 |
| Recreational area | 5 | 4.83 | 5 | 3.5 | 4 | 3.83 | 5 | 2.5 | 5 | 4.76 | 5 | 4.25 |
| Layout | 5 | 4.8 | 5 | 3.33 | 3.67 | 3.69 | 5 | 2.33 | 4.67 | 4.74 | 5 | 4 |
| Lighting | 5 | 4.86 | 5 | 2.5 | 4 | 4.07 | 5 | 2.5 | 5 | 4.64 | 5 | 3.5 |
| Distance | 5 | 4.79 | 5 | 3 | 4 | 3.74 | 5 | 2 | 5 | 4.74 | 5 | 3.5 |
| Functional facilities | 5 | 4.64 | 5 | 2.75 | 4 | 3.71 | 5 | 2.25 | 4.75 | 4.45 | 5 | 2.5 |
| Accessibility | 5 | 4.76 | 5 | 3.25 | 3 | 3.04 | 4 | 1.75 | 5 | 4.62 | 5 | 3.75 |
| Privacy | 5 | 4.52 | 5 | 2 | 3.33 | 3.23 | 5 | 1 | 4.67 | 4.57 | 5 | 1.33 |
| Indoor Environment | 5 | 4.75 | 5 | 3.5 | 4 | 3.74 | 5 | 2.25 | 4.75 | 4.72 | 5 | 4 |

Note: SI = Social Interaction; BE = Built Environment; *Med* = median; *Mean* = arithmetic mean; *Max* = maximum observed value; *Min* = minimum observed value.

Table 3 Coefficient–significance matrix of all predictor

| Predictor | SI1 | SI2 | SI3 | SI4 |
| --- | --- | --- | --- | --- |
| BE1-Space | • | • | • | • |
| BE2-Recreational area | • | • | • | **--** |
| BE3-Layout | • | • | • | • |
| BE4-Lighting | • | • | • | - |
| BE5-Distance | • | • | • | • |
| BE6-Functional facilities | **++** | • | • | • |
| BE7-Accessibility | • | **++** | **++** | • |
| BE8-Privacy | **-** | • | • | • |
| BE9-Indoor Environment | • | • | • | • |

**Note**: ++ p < 0.01 (positive), + p < 0.05 (positive), • not significant, - p < 0.05 (negative), -- p < 0.01 (negative)

Table 4 SEM structural paths with effect sizes (95% CIs) and FDR-adjusted q-values

| **Path** | **Std.β** | **95% CI** | **p** | **q (FDR)** |
| --- | --- | --- | --- | --- |
| SI1 ← BE6 | .585 | [0.260, 0.910] | P < .001 | q < .002 |
| SI1 ← BE8 | -.078 | [−0.313, 0.157] | P = .517 | q = .517 |
| SI2 ← BE7 | .541 | [0.288, 0.794] | p < .001 | q < .001 |
| SI3 ← BE7 | .459 | [0.271, 0.647] | p < .001 | q < .001 |
| SI4 ← BE2 | -.129 | [−0.325, 0.067] | p=.196 | q = .196 |
| SI4 ← BE4 | -.339 | [−0.576, −0.102] | p = 0.005 | q = 0.005 |

**Notes:** Paths are structural relations among latent variables. Effect sizes are Estimates with 95% Wald CIs, and Std. β are provided for comparability. Benjamini–Hochberg FDR was applied within each SEM model across structural paths; q-values are reported next to raw *p*-values. CI and FDR adjustments are used for interpretation rather than star thresholds.

Table 5 Model diagnostics for linearity, normality, heteroskedasticity (Glejser), and multicollinearity (Max VIF) for Models 1–4

| **Model** | **Shapiro–Wilk p** | **Glejser p** | **Max VIF** |
| --- | --- | --- | --- |
| 1 | .005 | .206 | 1.151 |
| 2 | .045 | .323 | 1.000 |
| 3 | .192 | .133 | 1.000 |
| 4 | .000 | .000 | 1.657 |

**Notes.** Shapiro–Wilk tests were computed on standardized residuals from each final model. Glejser p-values come from regressions of |residuals| on the predictors in each final specification (Enter method). Max VIF values are taken from the main regression models (Table 5). Values of 0.000 are reported as < .001.


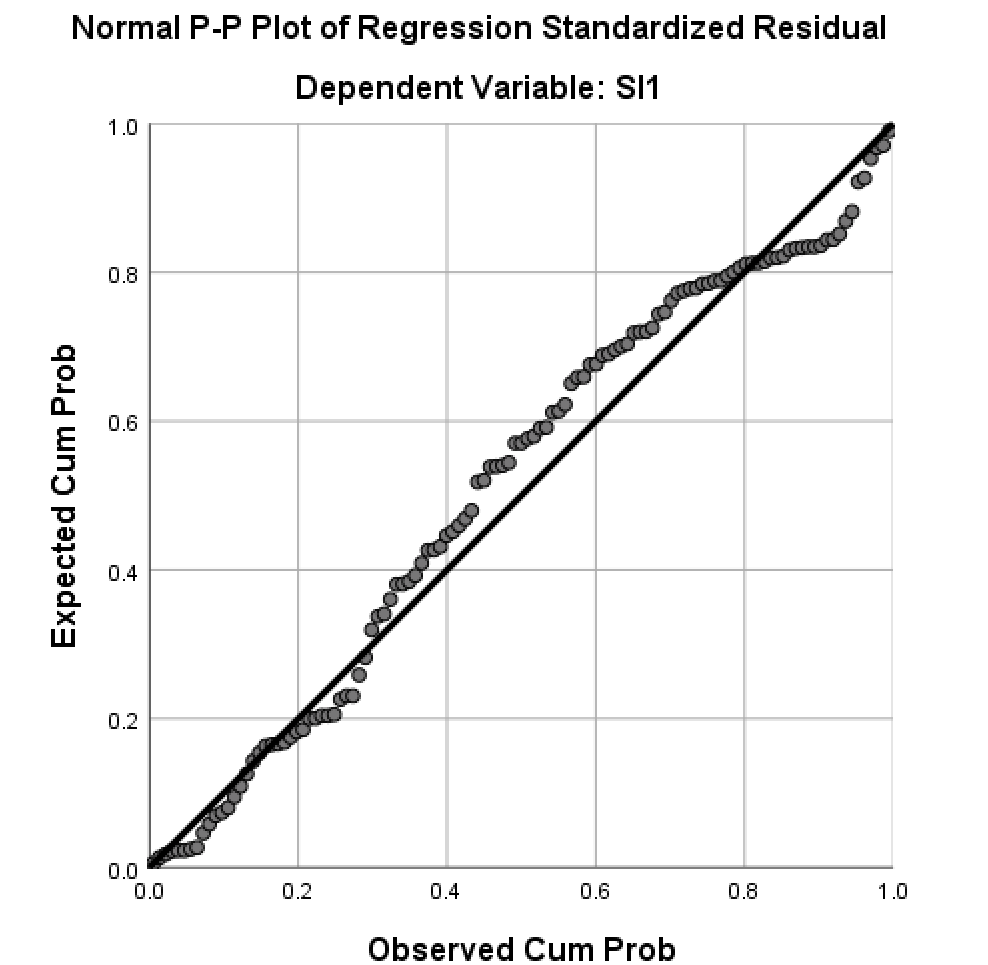

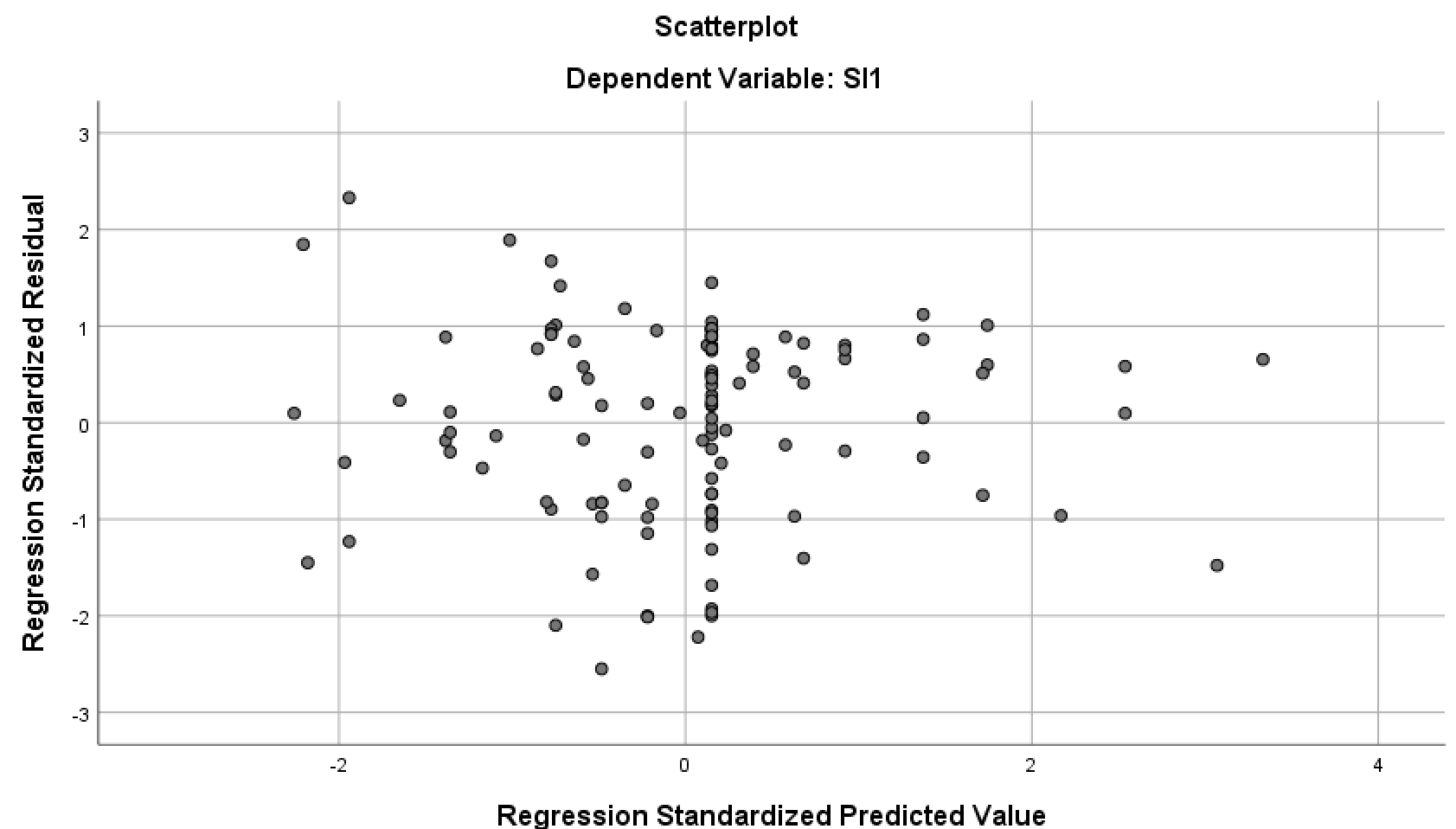


Figure 2a Model 1 (SI1) diagnostics: P–P plot and residuals–fitted scatter.


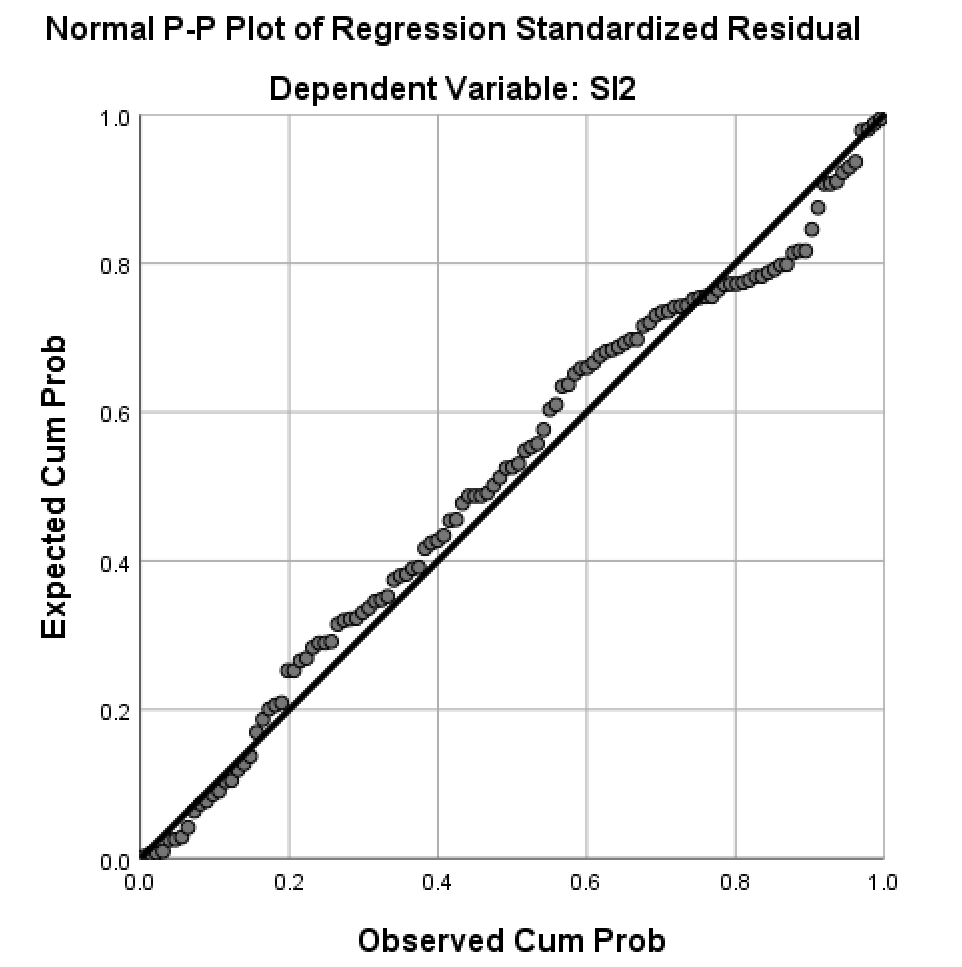

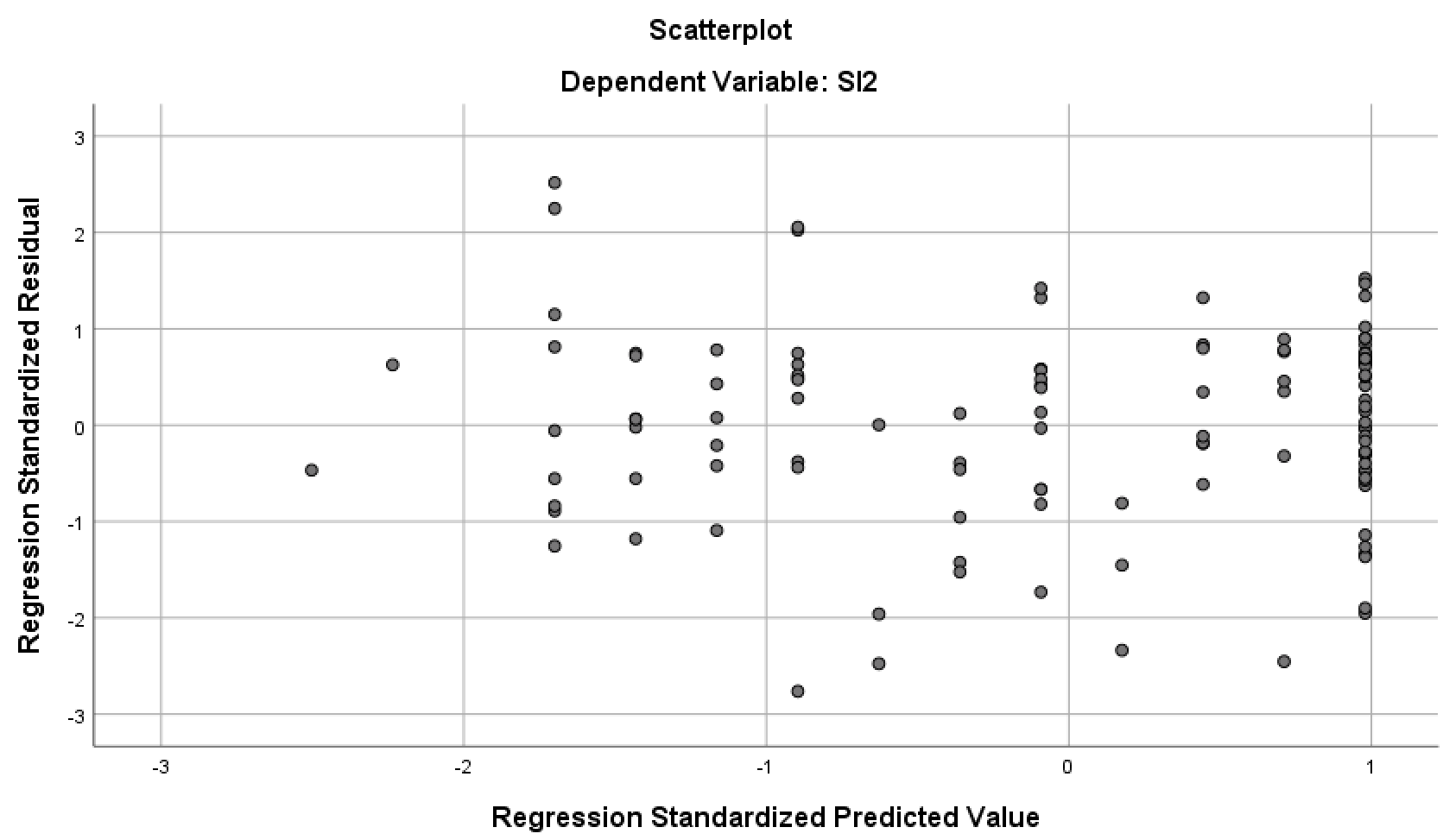


Figure 2b Model 2 (SI2) diagnostics: P–P plot and residuals–fitted scatter.


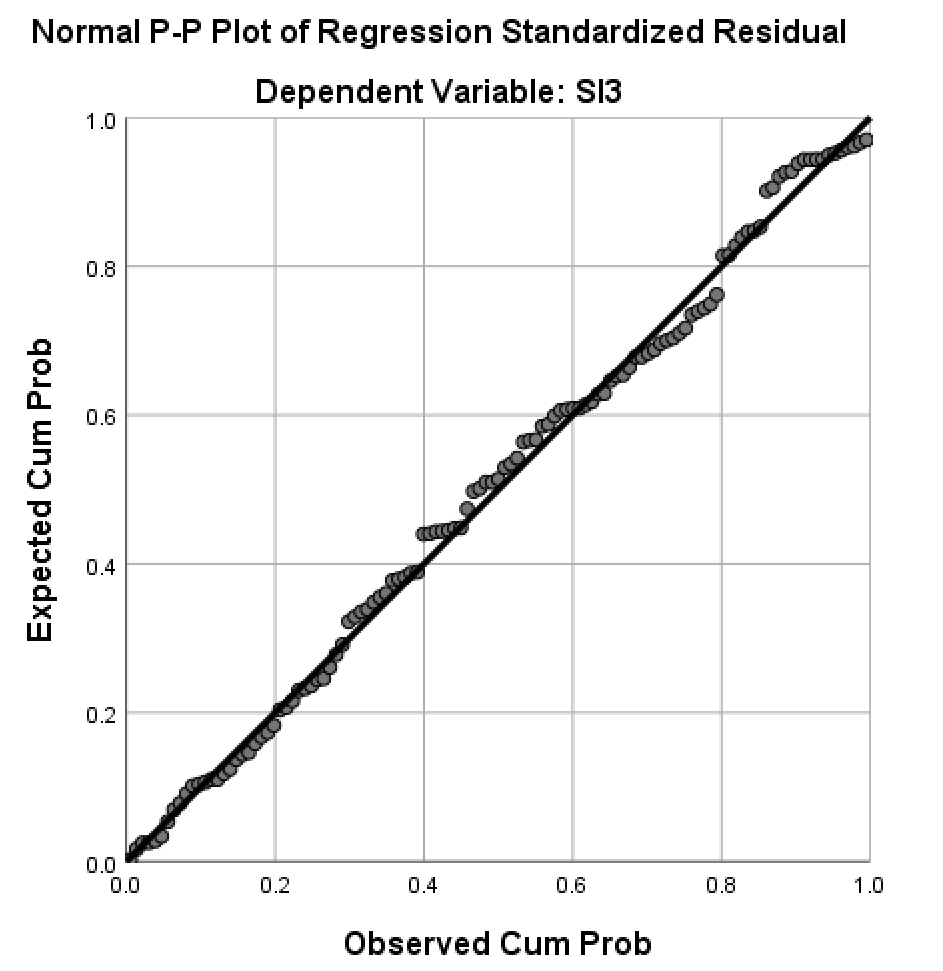

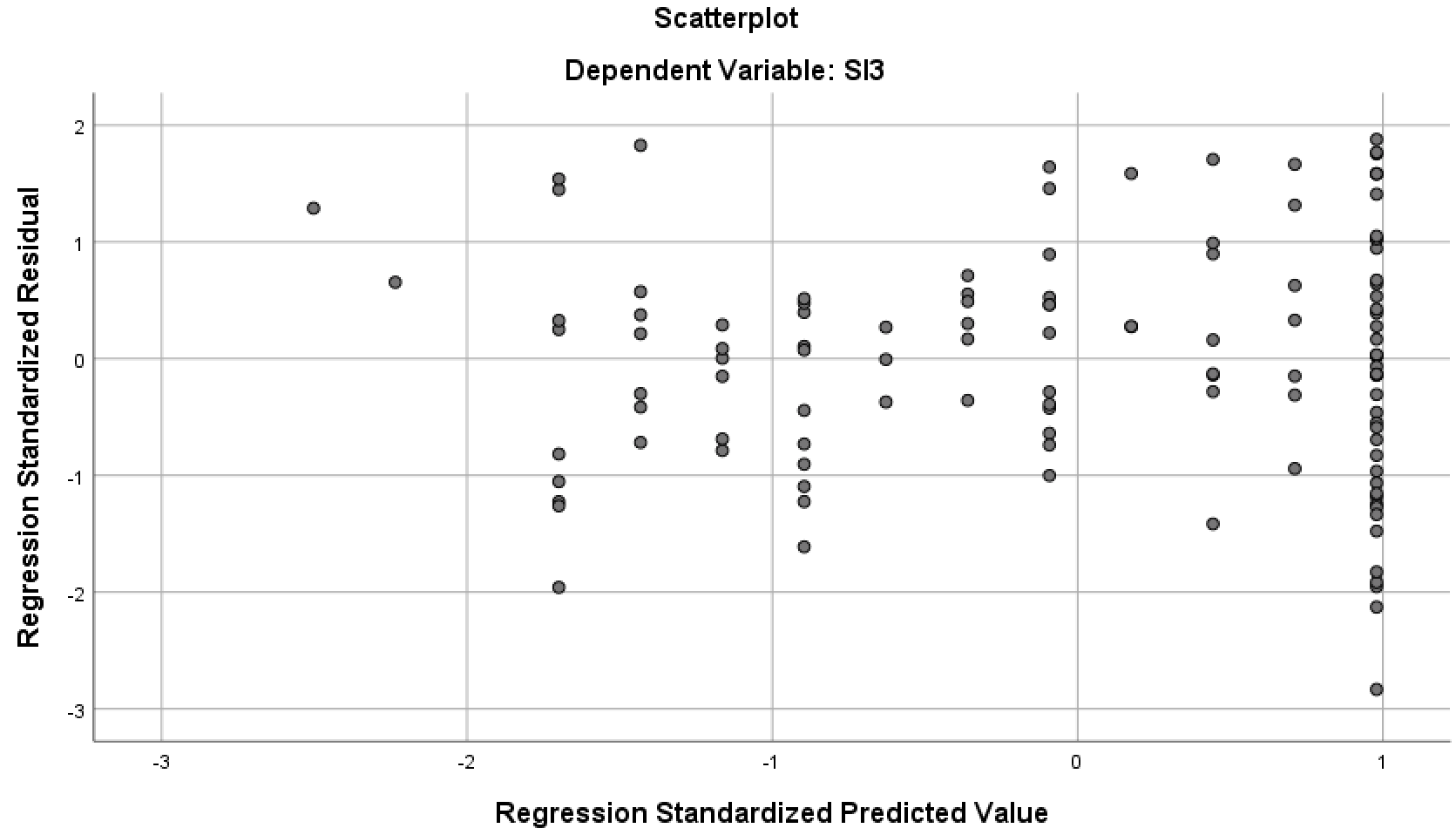


Figure 2c Model 3 (SI3) diagnostics: P–P plot and residuals–fitted scatter.


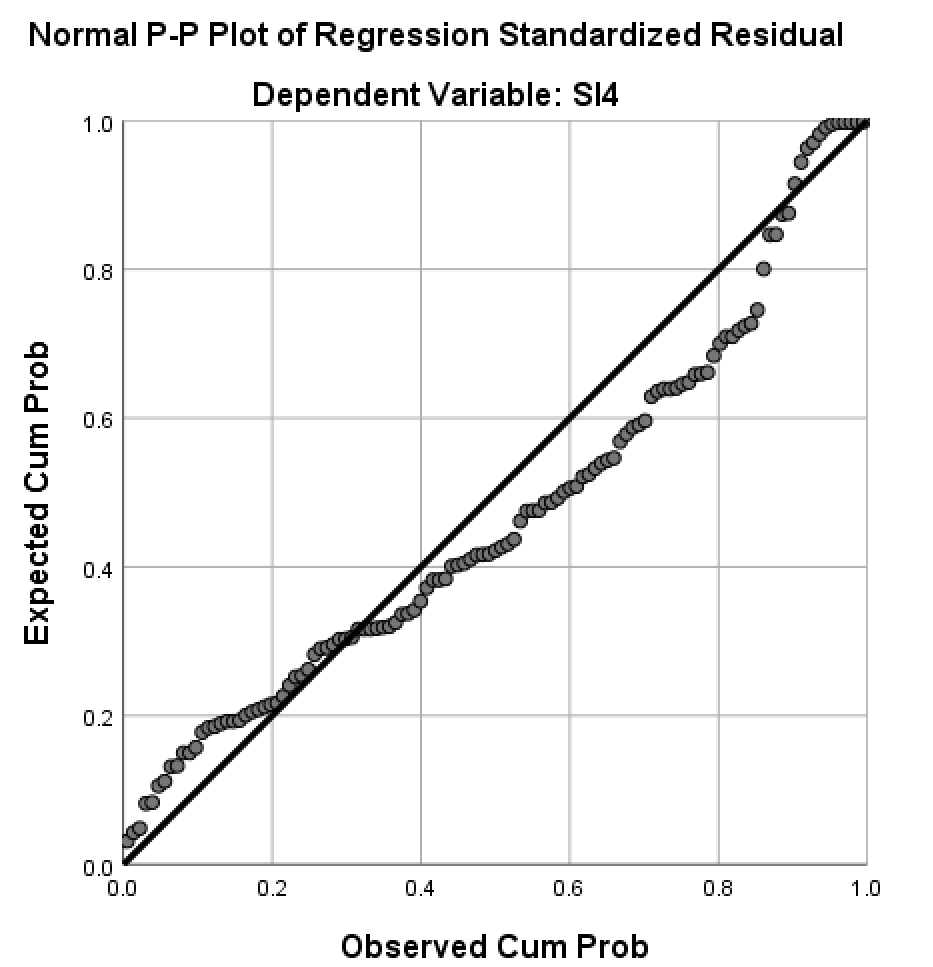

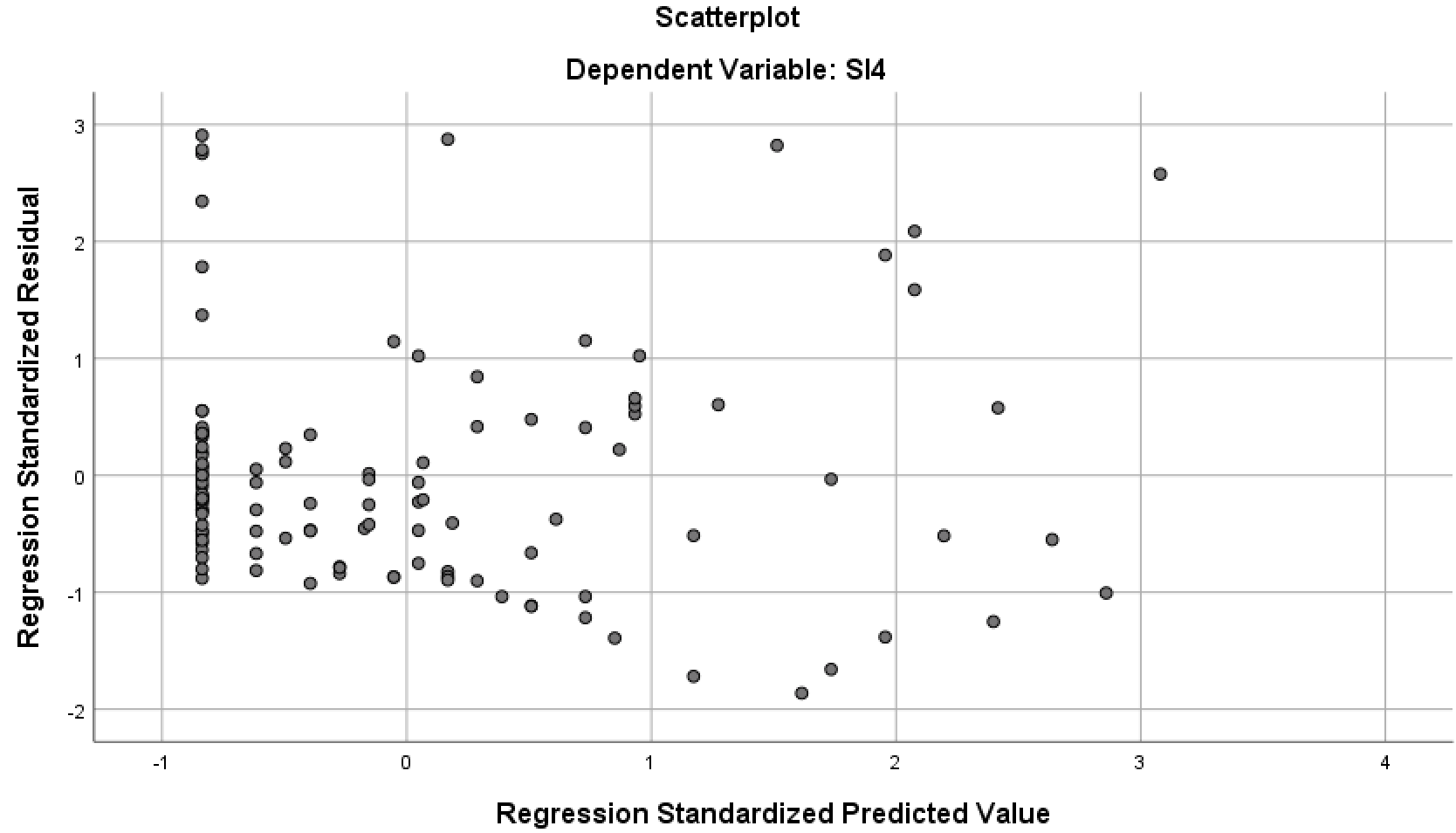


Figure 2d Model 4 (SI4) diagnostics: P–P plot and residuals–fitted scatter.
